# Organ-resolved lipid mapping in *Steatoda nobilis* spider model using high-resolution mass spectrometry imaging and Kendrick mass defect analysis

**DOI:** 10.3389/fchem.2025.1658546

**Published:** 2025-09-03

**Authors:** Damien Redureau, J. P. Dunbar, Raphaël La Rocca, Axel De Monts De Savasse, Quentin Bastiaens, Virginie Bertrand, Christopher Kune, Mathieu Tiquet, Johann Far, Edwin De Pauw, Michel M. Dugon, Loïc Quinton

**Affiliations:** 1 Mass Spectrometry Laboratory, MolSys RU, University of Liège, Liège, Belgium; 2 Venom Systems and Proteomics Lab, School of Natural Sciences, Ryan Institute, University of Galway, Galway, Ireland; 3 Midlands Bug and Reptile Zoo, Longford, Ireland

**Keywords:** lipidomics, lipids/chemistry, mass spectrometry imaging, Kendrick mass defects, whole-body imaging, arachnid

## Abstract

The noble false widow spider (*Steatoda nobilis*), a rapidly spreading member of the Theridiidae family, has gained attention for its increasing presence near human habitats and its medical significance due to envenomation reports. Recent studies have revealed that its venom contains α-latrotoxins, toxins also found in *Latrodectus* (black widows), responsible for latrodectism symptoms. Despite this growing interest, little is known about the lipidome and metabolome of *S. nobilis*, which could offer insights into its ecological role, dietary metabolism, and chemical communication. In this study, we used Matrix-Assisted Laser Desorption/Ionization Fourier-Transform Ion Cyclotron Resonance (MALDI-FT-ICR) mass spectrometry imaging (MSI) to investigate the whole-body lipid and metabolite distribution in *S. nobilis*. MSI is a powerful tool that couples molecular analysis with spatial information, enabling detailed visualization of biomolecules in tissues. Applying MSI to arachnids offers a novel approach to explore organ-specific metabolic profiles and identify potentially bioactive or adaptive compounds. One of the major challenges was preserving the spider’s fragile internal anatomy during sample preparation. We developed a gelatin-based fixation method to obtain intact histological sections suitable for MSI analysis. This allowed us to clearly distinguish organ-specific lipid and metabolite distributions *in situ*, including within the silk glands, ovaries, and nervous tissues. A second challenge was managing the vast data generated by MSI, with each image yielding thousands of molecular peaks. To streamline analysis, we employed Kendrick Mass Defect (KMD) plots to classify ions into structural families. This approach enabled us to link specific ions to molecular families and localize them within the spider’s body, enhancing our anatomical understanding at the molecular level. This work not only provides foundational insights into *S. nobilis* biochemistry but also demonstrates the potential of MSI for advancing arachnid lipidomics and uncovering molecules of ecological or biomedical interest. It opens the gates for broader applications of spatial lipidomics in other small biosystems and animals, particularly those previously inaccessible to detailed biochemical analysis.

## Graphical Abstract

**Figure:**
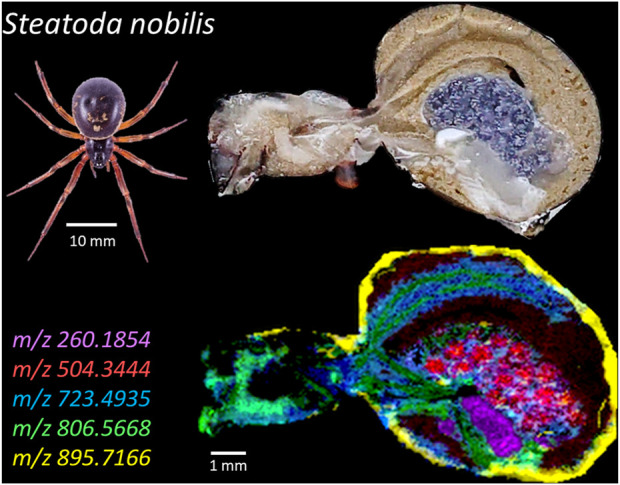

## Introduction

Mass spectrometry has been applied to elucidate the lipidome and metabolome of various biological systems, but the world of arachnids has been mostly unexplored. Arachnids, a class of chelicerate arthropods characterized by eight legs and a body divided into two tagmata, represent a fascinating group of organisms with unique physiological adaptations and ecological roles (Stork, 2017). This class encompasses a wide variety of organisms, including spiders, scorpions, ticks, and mites. With approximately 120,000 species described, arachnids occupy a broad range of ecological niches, displaying remarkable adaptations to different environments and lifestyles. As predatory arthropods, arachnids play essential roles in controlling insect populations, maintaining ecosystem balance, and serving as indicators of environmental health (Marc et al., 1999). Within the Class Arachnida, spiders (Order Araneae) produce venoms mainly used for predation and defense. Venom composition is highly diverse and contains a complex mixture of proteins, peptides, and small molecules, such as metabolites (Langenegger et al., 2019). Most of the current studies target the peptide and protein content of venoms, looking for novel pharmacological tools and potential future drugs. In this context, spider venom lipidome and metabolome have been poorly considered (Chen et al., 2023; Klupczynska et al., 2018), even if these could provide insights into the envenoming process. Spiders are also renowned for their silk production, which serves multiple purposes, including prey capture and the construction of shelters, brooding chambers and egg sacs (Römer and Scheibel, 2008). While extensive research has focused on the composition (Lu et al., 2024) and gene expression (Connor et al., 2024) of silk proteins, relatively little attention has been given to the underlying metabolic pathways that support silk biosynthesis. Lastly, spiders often experience long periods of fasting and have therefore evolved metabolic mechanisms to store and mobilize energy reserves very efficiently, which can be investigated again through lipidomics and, at a lower level, metabolomics.

### Steatoda nobilis, our model for whole-body MSI in lipidomics

We chose the noble false widow spider *Steatoda nobilis,* native to the Canary Islands and Madeira, as a model organism. The species is now well-established and invasive in parts of the Americas and Western Europe, particularly the United Kingdom, Ireland, and increasingly throughout western continental Europe due to a range of adaptive traits after spreading using global trade routes (Dugon et al., 2017; Rayner et al., 2022). Its success in colonizing new environments has raised ecological and public health concerns (Dunbar et al., 2018; Dunbar et al., 2022b). Morphologically, *S. nobilis* is a medium-sized spider, with females reaching up to 15 mm in body length and over 30 mm in leg span (Supplementary Figure S1). *S. nobilis* presents a dark brown to black bulbous abdomen with a distinctive pale cream or ivory-colored pattern that may resemble a skull or hexagonal geometric motif. Like all other spiders, *S. nobilis* possesses a cephalothorax and an abdomen, connected by a narrow pedicel, eight jointed legs protruding from the cephalothorax, prominent spinnerets on the posterior aspect of the abdomen, and anterior chelicerae from which venom is secreted. Internally, spiders feature a simple digestive system, book lungs, silk glands, a pair of large venom glands extending within the cephalothorax and connected to the chelicerae, a prominent curved heart and a nervous system adapted for efficient prey capture and environmental sensing. In addition, females possess an ovary sac, an oviduct and a sperm receptacle used for fecundation (Supplementary Figure S2). While the detailed internal anatomy of *S. nobilis* has never been investigated, main morphological characters are generally conserved across spiders, with minor variations occurring (e.g., number of eyes, number and types of silk glands). A notable exception is the occurrence of complex, highly variable and species-specific genitalia of both males and females. Considering their close phylogenetic relatedness, it is highly likely that the internal anatomy of *S. nobilis* is almost identical to the one of Black widows of the genus Latrodectus, which have been studied into more details (Smith et al., 1969; Whitehead and Rempel, 1959).

The diet of *S. nobilis* includes insects, other spiders, and occasionally small vertebrates, which raises concerns over potential ecological impacts on native fauna (Dugon et al., 2023; Dunbar J. P. et al., 2018; Dunbar et al., 2022a). *S. nobilis* venom has been studied at the proteotranscriptomic level, demonstrating the presence of α-latrotoxins and, all of which are also present in the venom of black widows. (Dunbar et al., 2020a). While the immediate medical threat posed by *S. nobilis* remains limited, public perception, its invasive status (Rayner et al., 2022) and its potential to disseminate antibiotic resistant bacteria (Dunbar et al., 2020b), justify further biological investigations. In that sense, studying the lipidome and the metabolome of *S. nobilis* may offer promising insights. As an emerging species of medical and ecological relevance, understanding its cuticular chemistry and organ-specific lipids could provide further insight into its adaptability, diet, and interactions with its environment. It could also help distinguish *S. nobilis* from native species at the molecular level, and reveal biochemical pathways linked to its success as an urban-adapted predator within its new geographical range.

### Metabolomics or lipidomics?

Metabolomics is a rapidly evolving field in the realm of systems biology that focuses on the comprehensive analysis of small bioorganic molecules, known as metabolites. Metabolites encompass a wide array of compounds, such as amino acids, lipids, sugars, and organic acids, which play critical roles in various biochemical pathways. Sometimes described as a subfield of metabolomics (Belhaj et al., 2021), lipidomics is increasingly recognized as a standalone field due to the complexity and functional diversity of lipids (Shevchenko and Simons, 2010). Lipidomics is defined as the large-scale study of pathways and networks of cellular lipids in biological systems including lipid composition, metabolism and function (Wenk, 2005). Together, metabolomics and lipidomics provide valuable insights into the dynamic molecular processes occurring within cells, tissues, and organisms, making it an essential tool for understanding the molecular underpinnings of physiological and pathological conditions (Wishart, 2019; Yang and Han, 2016).

### Mass spectrometry and liquid chromatography as gold standard techniques in lipidomics

Mass spectrometry (MS) has emerged as one of the most powerful and versatile analytical techniques for molecular research (Alseekh et al., 2021). MS allows for the accurate determination of the mass-to-charge ratio (*m/z*) of various ions, providing information about the elemental composition (high-resolution mass spectrometry) and structure of molecules (tandem mass spectrometry or MS/MS). In the context of lipidomics, MS is particularly valuable due to its ability to work in negative and positive polarities, to detect a wide range of lipids, from small one (fatty acids) to larger ones (triglycerides, ceramides …), with high sensitivity and selectivity. Tandem mass spectrometry is intensively exploited to elucidate with increased certainty, structures of molecules and even isomers, a real bottleneck in lipidomics.

Liquid chromatography (LC)-MS/MS is widely used in lipidomics, and more globally in metabolomics, because it offers high sensitivity, excellent selectivity, and the ability to separate and identify a broad range of metabolites in complex biological samples, even polar and large ones.(Hajnajafi and Iqbal, 2025). However, a major drawback of LC-MS/MS is that it requires extensive sample preparation and extraction, which destroys the spatial distribution of molecules within each organ. As a result, information about their localization is lost. This limitation is overcome by mass spectrometry imaging (MSI), which allows for the direct, label-free visualization of molecular distributions across tissue sections while preserving spatial information(Abu Sammour et al., 2023).

### Insights gained from mass spectrometry imaging in that context

Focusing on arthropods, MSI has been used to analyze metabolites in insect tissues, enabling visualization of the composition, abundance, and spatial distribution of endogenous and exogenous metabolites (Yang et al., 2020). MSI has additionally been employed for the rapid and accurate identification of various arthropod species, including mosquitoes and ticks, which are all vectors of human diseases (Sánchez-Juanes et al., 2022). Furthermore, MSI has been applied to map the architecture of animal toxin systems, generating molecular images at the micrometer scale to spatially map toxin compositions in producing organisms (Damm et al., 2025). Although full body MS imaging has already been proposed for rat pharmacokinetics (Stoeckli et al., 2007) and for methodological aims on zebrafish (Ma et al., 2022; Stutts et al., 2020), the knowledge about organ-specific lipidome and metabolome of other animals, including spiders, is very limited (Khalil et al., 2015; Khalil et al., 2017). This is mainly because the size of most spiders represents a significant bottleneck, but also because obtaining traditional histological sections from mid-sized spiders such as *Steatoda* presents significant challenges due to their unique anatomy and tissue organization.

Indeed, unlike vertebrates, whose bodies contain large, soft internal organs embedded in tissue-rich matrices, spiders possess a compact and highly specialized body plan dominated by non-cellular structures and fluid-filled cavities (Foelix, 2010; Propistsova et al., 2023). Indeed, the exoskeleton of spiders is made of chitin, a hard and chemically resistant polysaccharide. This rigid outer cuticle encases most of the body and acts as a protective shell, making it difficult for fixatives such as formalin to penetrate the tissues uniformly. As a result, internal structures may not be adequately preserved during the slicing, leading to poor morphology or tissue degradation during processing (Polilov et al., 2021). Secondly, the internal anatomy of spiders is not dominated by solid tissues but rather by the hemocoel, a large cavity filled with hemolymph. Most internal organs are suspended within this cavity and are often fragile or loosely organized (Supplementary Figure S2). Another complicating factor is the small size and dense packing of functionally diverse organs within the cephalothorax and abdomen. Organs are often intertwined or stacked closely together with minimal connective tissue separation, making it difficult to isolate specific systems in sections or to orient the specimen predictably (Propistsova et al., 2023). Finally, spiders have numerous air-filled tracheae and book lungs (2 or 4), which can easily collapse. These structures are difficult to preserve in a way that maintains their three-dimensional architecture, and their presence often leads to inconsistent sectioning and tissue fragmentation. Although previous studies have established methods for fixative infusion through the cuticle of decapod crustacean larvae to preserve internal organ architecture prior to sectioning (Torres et al., 2021), applying such approaches to spiders may prove more challenging due to their more flexible and resistant cuticle. For these reasons, the first part of the study focuses on the sample preparation strategy we employed to get histological slices suitable for mass spectrometry imaging.

From another perspective, a key strength of MSI lies in its ability to analyze organs at the molecular level without micrometer-scale laser microdissection, preserving anatomical context and revealing lipidomes within structures like silk glands or the digestive tract. MSI resolution is crucial for the quality and interpretability of spatial molecular data. For a spider around 1 cm long, the number of pixels depends on the lateral resolution: at 100 μm, ∼10,000 pixels provide a general organ-level view; at 10 μm, over 1 million pixels can reveal cellular or glandular features. While higher resolution offers finer molecular maps, it also increases acquisition time, data size, and computational load. Thus, selecting resolution involves balancing spatial detail with data throughput, especially for small, densely packed organisms like spiders.

Moreover, each pixel is associated with a full mass spectrum, made of thousands of peaks, increasing even more the complexity of the datasets that finally require advanced computational tools for interpretation. Data processing appears then as a major bottleneck in MSI studies (Krijnen et al., 2024). Reducing the difficulty of data treatment becomes highly appealing. Strategies such as data compression, region-of-interest (ROI) selection, segmentation or machine learning-based feature extraction help simplify analysis without compromising biological insight (Burguet et al., 2024; La Rocca et al., 2021). Streamlining data workflows not only accelerate interpretation but also makes high-resolution MSI more accessible and scalable, especially when applied to intricate samples like whole-body spider imaging.

Moreover, given the molecular diversity detected across different organs and tissues, traditional peak annotation can be challenging even with the help of exact mass measurements and various databases of metabolites. Kendrick Mass Defect (KMD) approach offers a particularly elegant solution for managing complex MSI data (Kune et al., 2019). This approach is based on a pioneering study made in 1963 by Edward Kendrick who proposed an identification of organic compounds based on the 2D projection of their atomic composition (Kendrick, 1963). KMD analysis enables the grouping of chemically related compounds (e.g., lipid series or metabolites) by aligning them based on repeating units (like CH_2_), simplifying spectral interpretation. As a result, KMD scatter plots, constructed by plotting the KMD in function of the *m/z*, becomes a powerful tool to reveal biochemical patterns and tissue-specific lipidomic and metabolomic signatures in spiders, even when reference databases are limited. Applied to MSI, this method allows researchers to identify molecular families and track their spatial distribution throughout the body.

In this study, we explore the use of mass spectrometry imaging to investigate the anatomy of *S. nobilis* based on its lipidome. The unique anatomical features of arachnids, such as their rigid exoskeleton, fluid-filled body cavities, and densely packed, delicate internal organs, pose significant challenges to traditional histological and molecular approaches. MSI, by enabling spatially resolved molecular analysis without the need for dissection or labelling, offers a promising alternative. However, imaging an entire organism, such as a 1 cm-long spider, at high resolution generates vast and complex datasets. Strategies that simplify data treatment, such as optimized resolution choices and dimensionality reduction, become essential. Notably, the integration of Kendrick Mass Defect (KMD) analysis further enhances interpretability by allowing the visualization and classification of molecular families across the body. Altogether, these methodological advances pave the way for a detailed, spatially resolved exploration of the spider’s organ-specific metabolome. More globally, the development of full-body mass spectrometry imaging (MSI) represents a significant advancement in the field of spatial lipidomics, enabling comprehensive, untargeted biochemical mapping across entire organisms. This approach opens new avenues for investigating the molecular architecture of small biosystems and animal models, particularly those that have historically been inaccessible to detailed biochemical analysis due to limitations in spatial resolution, sample size, or tissue complexity.

## Materials and methods

### Spider sourcing

*S. nobilis* specimens were collected at night on street furniture (e.g., metal fences, stone walls) and tree trunks in the suburban areas of County Dublin in Ireland, using forceps and 50 mL Falcon tubes. Once collected, specimens were maintained at room temperature and lightly misted with water every 2 days until being processed.

### Sample preparation

Spiders do not behave like homogeneous, solid biological tissues during freezing, which makes standard cryosectioning techniques extremely challenging. To overcome these issues, one solution is to perform whole-body cryoembedding followed by careful cryosectioning at low temperatures, which preserves delicate structures better. Traditionally, biological frozen tissues are embedded in tissue Tek OCT (Optimal cutting temperature), a soluble resin matrix often used for the imaging of individual organs. This approach is however not suitable for spiders due to their hard exoskeletons and the lack of internal support structure. It additionally may disrupt the MS spectra by contaminating the samples and adding polymer-like signals that may mask metabolite ions. Ice embedding has also been considered but does not stabilize the spider body enough. Ice is very fragile during sectioning as well and tends to chip with hard structures like cuticles. Instead, we have chosen to work with gelatin embedding because it represents an excellent compromise between structural preservation and compatibility with downstream MS analyses. Live specimens were anesthetized using a CO2 chamber, incorporated into melted gelatin 20% w/v. Gelatin powder (VWR 24350.262, CAS 9000-70-8) was dissolved in ultrapure water by heating the solution to 40 °C under continuous stirring using a magnetic stirrer), flash-frozen in isopentane (Sigma-Aldrich, CAS 78-78-4) pre-cooled with liquid nitrogen. Frozen gelatin blocks were mounted in a cryostat set between −18 °C and −20 °C and sectioned at a thickness of 14 µm. Slicing parameters are the following: blade angle (10°–15°), temperature (−10 °C to −15 °C). The obtained sections were then directly transferred either to classical slides for standard histological staining or to indium tin oxide (ITO)-coated conductive glass slides (Bruker Daltonics), dedicated to MSI analysis. To visualize the tissue topology and assist in the anatomical assignment of mass spectrometry signals, hematoxylin and eosin staining was performed on cryosections serial to those used in MSI analysis. Slides underwent sequential rehydration in ethanol and water: absolute ethanol (4s), 90% ethanol (4s), 70% ethanol (4s), and distilled water (4s). Samples were then stained in Gill III hematoxylin for 2 min, followed by 0.1% HCl dip (2s), rinsing under running tap water (3 min), eosin staining (1 min), distilled water rinse (4s), and dehydration through ascending ethanol series: 70% ethanol (4s), 90% ethanol (4s), and absolute ethanol (4s). For MSI, the MALDI matrix was applied using the SunCollect (SunChrom, Germany) with the Z-axis set to its minimum height to ensure optimal deposition on the spider surface. A solution of α-cyano-4-hydroxycinnamic acid (CHCA) at 5 mg/mL in acetonitrile/water/trifluoroacetic acid (ACN/H_2_O/TFA, 70:29.9:0.1, v/v/v) was used. The sprayer operated with an X:Y stage speed of 1540 mm/min. A total of 12 matrix layers were deposited with a progressive flow rate: the first layer at 5 μL/min, the second at 10 μL/min, the third at 15 μL/min, and layers four through twelve at 20 μL/min. This gradual increase in flow rate ensured homogeneous matrix coverage and minimized analyte delocalization.

### Mass spectrometry imaging

MSI data were acquired on a ESI/MALDI solariX xR 9.4T instrument (Bruker Daltonics, Bremen, Germany) using a mass range of 200–1400 m*/z* in amplitude mode and a file size of 2M time domain. This FT-ICR mass spectrometer is fitted with a high field supraconductive magnet, dynamically harmonized ICR (ion cyclotron resonance) cell, and 1ω detection capable of extreme mass resolving power (FWMH of minimum 1 million at *m*/*z* 400) and an expected mass accuracy often better than 1 ppm). In MSI experiments, such performances are hardly met due to space charge, even in FT-ICR Mass Spectrometry (McCann et al., 2021), or again the constraint imposed by the file size. In the current study, the targeted conditions were a FWMH ±300,000 @ 400 *m*/*z*, and an accuracy of 3 ppm. For each mass spectrum (pixel) of the image, the MALDI laser beam focus was set to “small” to reach a lateral resolution of the pixel’s image around 50 μm. Twenty laser shots were performed at a repetition rate of 200 Hz on each pixel. The laser power was tuned to produce a stable ion current between pixels as described elsewhere (Tiquet et al., 2022). The mass spectrometer was externally mass calibrated from 200 m/*z* to 1400 m/*z* using red phosphorus suspension in pure acetone and spotted directly onto the slides. The laser power was tuned to produce a similar number of ions than those obtained from the sample. FlexImaging v5 (Bruker Daltonics, Bremen, Germany) software was used to design the imaged areas for MALDI-MSI acquisition (outlines of the spider), using a pixel step size for the surface raster set to 50 μm.

### Data analysis

Imaging raw data files were first exported as imzML files (i.e., standard open format for MS file (Schramm et al., 2012)). MSI data was recalibrated (in the m/z dimension) using the method described by La Rocca and colleagues (La Rocca et al., 2021) La Rocca et al. The recalibration function was applied with the following parameters: st 0.0003, -tl 0.02, and -lm 0.002. Prior to recalibration, the MSI dataset was exported in centroid format and submitted to METASPACE for annotation using the following databases: HMDB v4, LIPID MAPS (2017-12-12), ChEBI (2018-01), and CoreMetabolome v3. A list of theoretical m/z values was compiled by selecting all annotated features with a false discovery rate (FDR) ≤ 10%, and these values were used as reference peaks for the recalibration process. After recalibration, the MSI data were re-submitted to METASPACE and annotated using the same databases and parameters. For Kendrick mass defect analysis, the mzML file was converted to ICK files and processed with our in-house Python software, MSKendrickFilter (MSKF) developed by Kune et al. (2019). Profile MS data was converted into centroid MS data using an implemented peak picking algorithm. An intensity threshold, a peak prominence, and a peak distance (number of data point between peaks) of, respectively, 200,000 cps, 0 and 5 were considered for this peak picking. Centroided MSI data was realigned to reduce centroid *m*/*z* value variations. Kendrick reference used in MSKF for lipids was -CH_2_-, and the Kendrick mass (KM) and Kendrick mass defect (KMD) were computed according to Equations 1, 2, where *m*/*z* is the measured mass-to-charge ratio of the ions using IUPAC scale.

$$KM=\frac{m}{z}.\frac{{KR}_{nominal\;mass}}{{KR}_{exact\;mass}}=\frac{m}{z}.\frac{14.00000}{14.01565}$$ (1)

$$KMD=round\left(KM\right)-KM$$ (2)

The program allows for different types of rounding for Equation 2: either rounding down to the nearest lower integer (KMD ranging from −1 to 0), rounding up to the nearest higher integer (KMD ranging from 0 to 1), or rounding to the nearest integer (KMD ranging from −0.5 to 0.5). In this work, rounding up was chosen as it provides a better distribution of lipid KMD values near the center of the KMD plot. The KMD filtering was applied to retain only the information linked with lipids of interest, by restricting the dataset to theoretical KMD values between 0.10 and 0.50 and m/z between 200 and 1,400. This range was chosen because it concentrates the majority of relevant lipid signals while minimizing noise and unrelated features. The MSKF software allows the metabolite and lipid signals to be grouped by classes, based on their KMD value (since ions varying only by -CH_2_- units have the same KMD value), with a given KMD tolerance. In the case where two or more lipid classes have a similar KMD value, a second algorithm is applied to group the lipid signals based on mass difference. This algorithm groups all ions whose mass difference corresponds to a given formula, here -CH_2_- with a given relative mass tolerance (2 ppm). It results in a set of potential lipid series that can be identified by database research. Moreover, MSKF allows for a simplification of the KMD plots by transforming the signal series coming from different isotopes or cation adducts of the same species into the protonated monoisotopic lipid series (Hustin et al., 2022).

### Database search and metabolite identification

Filtered data were analyzed by MSKF to isolate the lipid classes and the metabolites present in the spiders. In a second round, the LIPID MAPS structure database (Conroy et al., 2024), METASPACE (Palmer et al., 2017) and Human Metabolome DataBase (Wishart et al., 2022) were interrogated for potential identification. The article strictly follows the lipid nomenclature defined by LIPID MAPS, adopting a structured and standardized classification of lipid categories, classes, and subclasses.

## Results

### Quality of the slices using gelatin embedment

As discussed in the introduction, spiders pose a challenge for histological slicing due to their rigid chitinous exoskeleton, small size, and internal anatomy composed largely of hemolymph-filled cavities with fragile and loosely organized organs. We have observed that the presence of hair on spider’s cuticle could prevent the embedding solution from making uniform contact with the entire surface of the sample, leading to poorly coated areas and potential air pockets, which in turn may cause distortions in the tissue sections.

**FIGURE 1:**
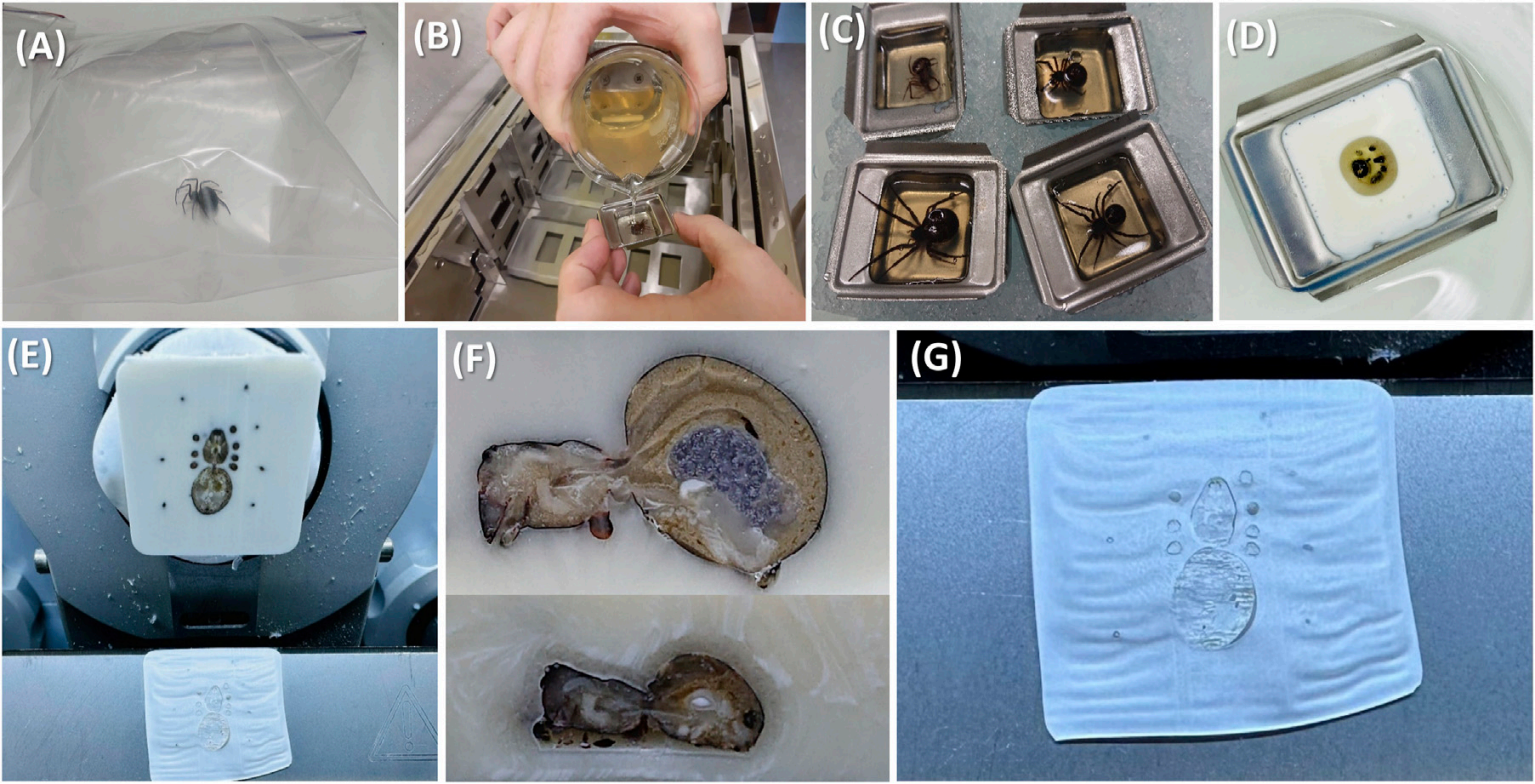
Sample preparation workflow allowing the production of histological spider slices suitable for MSI. **(A)** Spiders are anesthetized using carbon dioxide. **(B)** Melted gelatin is poured over the spider, then **(C)** kept on ice. **(D)** The gelatin is solidified using a bath of isopropanol cooled down with liquid nitrogen. **(E)** The embedded spider is cut in thin slices (14 µm) using a cryostat. **(F)** Female (top) and male (bottom) anatomy can already be discovered at the histological level. MSI aims to provide a molecular view of these structures. **(G)** Slices are ready to be covered by the suitable MALDI matrix and introduced in the mass spectrometer.

Instead of traditional methodologies, we have chosen to work with gelatin embedding because it represents an excellent compromise between structural preservation and compatibility with downstream MS analyses. To obtain high-quality anatomical sections of *S. nobilis* suitable for mass spectrometry imaging (MSI), we developed a protocol combining gentle anesthesia, cryoembedding, and controlled sectioning (Figure 1). The procedure begins with the anesthesia of live spiders using carbon dioxide (Figure 1A). Individual spiders were placed in small transparent plastic bags and exposed to a CO_2_ flow for a few minutes until complete immobilization. This method provided a quick (and reversible) anesthetic effect, minimizing stress and preserving tissue integrity. Once anesthetized, the spiders were positioned in small metal embedding molds containing a thin layer of melted gelatin. Melted gelatin (10%–20%) was subsequently poured over each specimen to embed their entire body (Figure 1B). The molds were kept on ice to ensure rapid solidification of the gelatin matrix while maintaining the anatomical posture of the specimen (Figure 1C). After the gelatin had sufficiently solidified, the entire mold was rapidly frozen by immersion in isopentane pre-cooled with liquid nitrogen (Figure 1D). This step ensures fast freezing while avoiding excessive ice crystal formation, which could compromise tissue morphology. The resulting frozen gelatin blocks were then mounted on the specimen holder of a cryostat typically set between −18 °C and −20 °C (Figure 1E). The embedded spiders were sectioned at a thickness of 14 μm, a compromise between anatomical visibility and MSI compatibility (Figure 1F). Sectioning was performed with careful adjustment of the blade angle, temperature, and cutting speed to accommodate both the rigid exoskeleton and the soft internal structures stabilized by the gelatin matrix. The blade angle was set between 10° and 15° to ensure optimal alignment with the sample surface. The cryostat chamber temperature was maintained between −10 °C and −15 °C, with the exact setting chosen based on the friability of the specimen. Cutting speed was adjusted manually during sectioning, slowing the feed when increased resistance was encountered (exoskeleton) to prevent tearing or compression of the internal organs and structures. The obtained sections, mostly preserving both the external morphology and internal organization of the spider body (Figure 1G), were then directly transferred either to classical slides for standard histological staining or to indium tin oxide (ITO)-coated conductive glass slides for downstream MSI acquisition.

**FIGURE 2:**
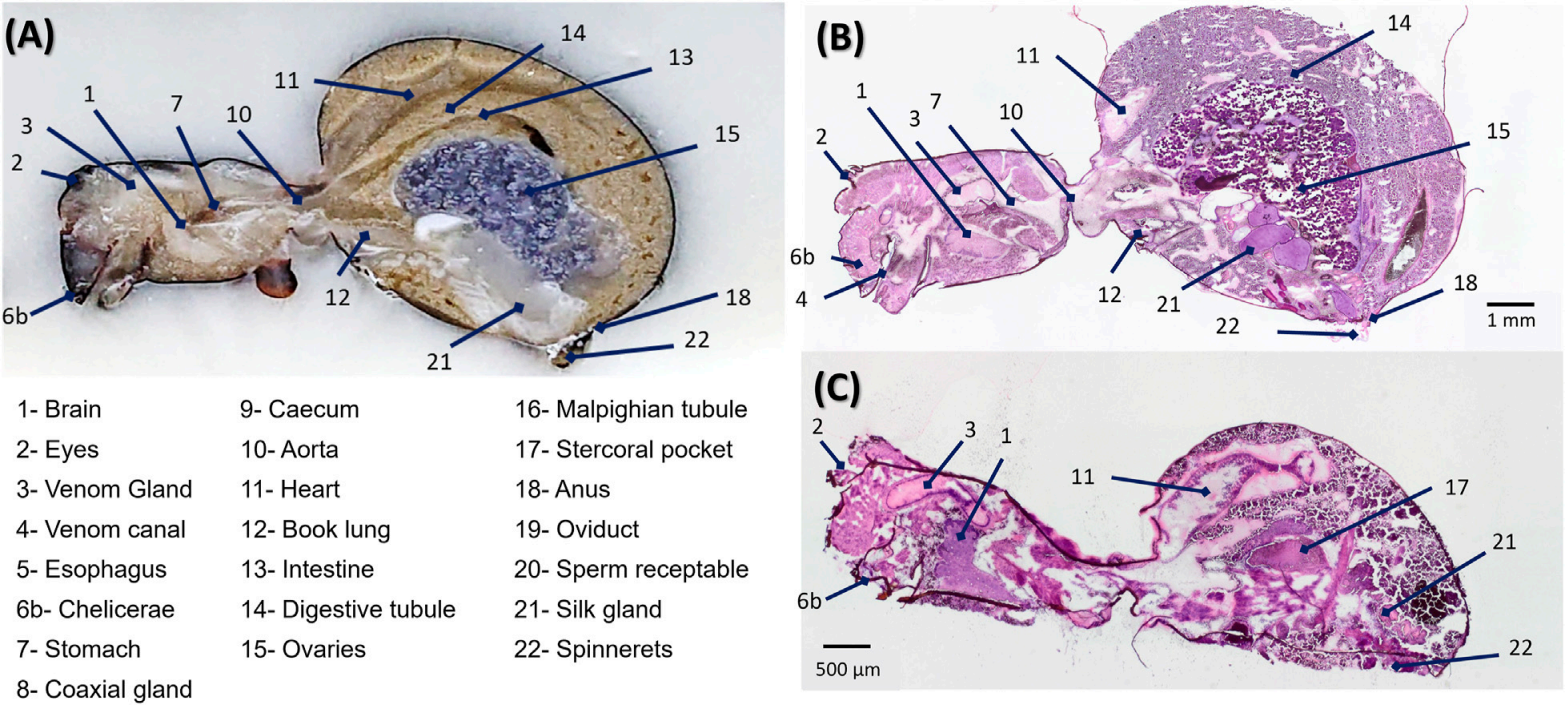
Internal anatomy of a female *Steatoda nobilis* spider. **(A)** Annotation of the internal organs within the prosoma and opisthosoma of an adult female *S. nobilis*. Major anatomical features include the central nervous system, digestive tract, silk glands, reproductive system, and circulatory system. By Ryan Wilson and Kimmel (2010). Histological staining of **(B)** female and **(C)** male specimen of *S. nobilis*. Incomplete organ identification can be made based on tissue morphology, especially for the smaller male, where the task is even more challenging.

The results of the histological staining performed on sagittal sections of both male and female *S. nobilis* specimens are presented in Figure 2. In female spiders, histological contrast was particularly effective in revealing the internal organization of the body (Figure 2B). Several anatomical regions, including the brain, digestive tubules, and reproductive structures such as the ovaries, were readily distinguishable and could be reliably annotated based on tissue morphology. In contrast, the histological interpretation of male spider sections proved more challenging. Owing to the male’s significantly smaller size, approximately half that of the female, its internal organs are more compact, making their localization and identification considerably more difficult (Figure 2C). This anatomical constraint limits the resolution and clarity of histological features in male sections. Although classical staining provides valuable anatomical insight, it offers limited information about tissue function. One of the aims of this study is to demonstrate that MSI can not only improve organ localization but also reveal organ-specific metabolic activity. Given the greater anatomical clarity and the presence of metabolically relevant female-specific organs, such as the ovaries, we chose to focus the remainder of the MSI analysis on female specimens exclusively.

The slides dedicated to mass spectrometry imaging were gently air-dried in a desiccator at room temperature to remove surface moisture while avoiding shrinkage or delocalization of analytes. The classical strategy for acquiring the image was then applied (see Supplementary Figure S3). To summarize, matrix application was performed with an automated spray-coating system (SunCollect, Sunchrom) to deposit a uniform layer of α-cyano-4-hydroxycinnamic acid (CHCA), a matrix suitable for MALDI analysis of small molecules and phospholipids. After matrix deposition, the slides were introduced into the MALDI-FT-ICR mass spectrometer (SolariX, Bruker) and a raster scan was configured over the tissue surface, with a laser spot size of 50 µm. Each pixel yielded a full mass spectrum, and thousands of spectra were recorded across the tissue surface, allowing for the reconstruction of ion distribution maps. Spectral data were processed using dedicated MSI software (SCiLS^©^), where ion images are typically generated for selected *m/z* values or processed by segmentation. The resulting molecular maps were overlaid on optical images of the section to allow direct anatomical correlation between chemical signals and internal structures of the spider.

### Data treatment by Kendrick mass defects

The high-resolution mass spectrometry imaging data acquired with MALDI-FT-ICR yielded rich spectral datasets containing thousands of *m/z* features distributed across the anatomical sections. Studying all these peaks one by one to extract biologically meaningful information is time consuming and poorly efficient. Dedicated computational tools, such as spectral alignment and normalization to ion image reconstruction, multivariate analysis, and molecular annotation, are then used to assist the researchers in this complex and challenging task.

While classical data analysis approaches such as unsupervised segmentation or principal component analysis (PCA) are effective for highlighting spatial heterogeneity across tissue sections, they often provide limited chemical interpretability, especially in complex, untargeted datasets. These methods cluster signals based on overall variance or spatial patterns, but do not inherently group chemically related species, making it challenging to distinguish molecular families or detect systematic modifications. In contrast, Kendrick Mass Defect (KMD) plots offer a chemically driven strategy that enables the recognition of homologous series and molecular families by normalizing mass values based on repeating units such as CH_2_, convenient for lipids. By reprojecting the data in KMD space [[KMD = f(*m/z*)], chemically related ions align along horizontal lines or clusters, even when their spatial localization is diffuse or variable. In this approach, identifying a single molecule within a homologous series enables the annotation of all related compounds aligned along the same horizontal line. This is possible because chemically related species share the same Kendrick mass defect, allowing for rapid and structure-informed propagation of identification across the dataset, assuming that molecules aligned horizontally (e.g., in a Kendrick mass defect plot) belong to the same structural lipid family, an assumption generally valid, though the propagation strategy should be applied with caution.

Each of the acquired MSI datasets was converted from the original “.imzML” format obtained from the acquisition software SCiLS (Bruker) into “.ick” files using the dedicated export tool integrated in the home made MSKendrickFilter (MSKF) software (*see Experimental Methods*). This step enabled visualization of the data in Kendrick Mass Defect (KMD) vs. *m/z* plots. The transformation was performed using CH_2_ as the base unit, a common strategy to align structurally related metabolites, particularly lipid series.

**FIGURE 3:**
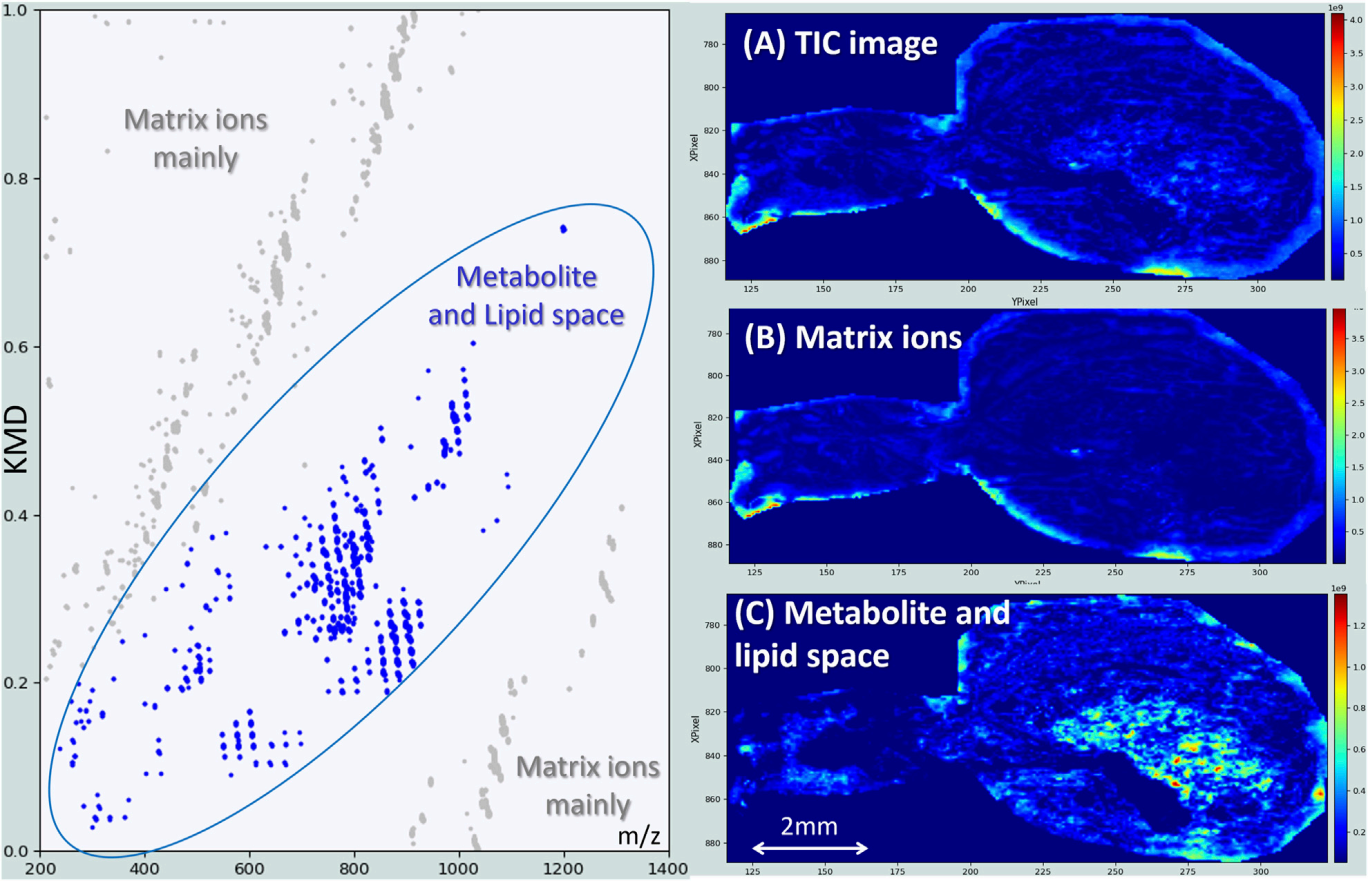
Kendrick’s mass defects plot obtained for the MSI of a female *S. nobilis*. When the contribution of the signals associated with matrix clusters [greys dots, image **(B)**] is removed from the Total Ion Current [greys and blue dots, image **(A)**], fine structures of spider anatomy clearly appear [image **(C)**]. The signals responsible for these structures are represented as blue dots.

The result obtained for a female *S. nobilis* is shown in Figure 3. In these plots, spectral peaks are represented as individual dots, which tend to cluster into distinct “clouds” or lines, reflecting families of chemically similar molecules. The image reconstructed from the total ion current (TIC) does not present any structure or contrast either (Figure 3A). This is mainly because the average spectrum is dominated by matrix-related ions present across large areas of the sample or even in background regions (Figure 3B). These undesired signals, however, do not align horizontally in our Kendrick’s plots, as it has been constructed with a CH_2_ base unit, which is not repeated in CHCA matrix ions. Therefore, they can be easily recognized due to their tendency to form characteristic and recurrent clusters and excluded from the signals used for image reconstruction (grey dots in the Kendrick plots of Figure 3). In the same way, lipids align very well and define a quite clear KMD-*m/z* space where most of the ions of interest will be grouped. The MS image constructed based on these signals only, already reveals the underlying structures of the spider anatomy (Figure 3C).

### KMD plots for the analysis of lipid groups

**FIGURE 4:**
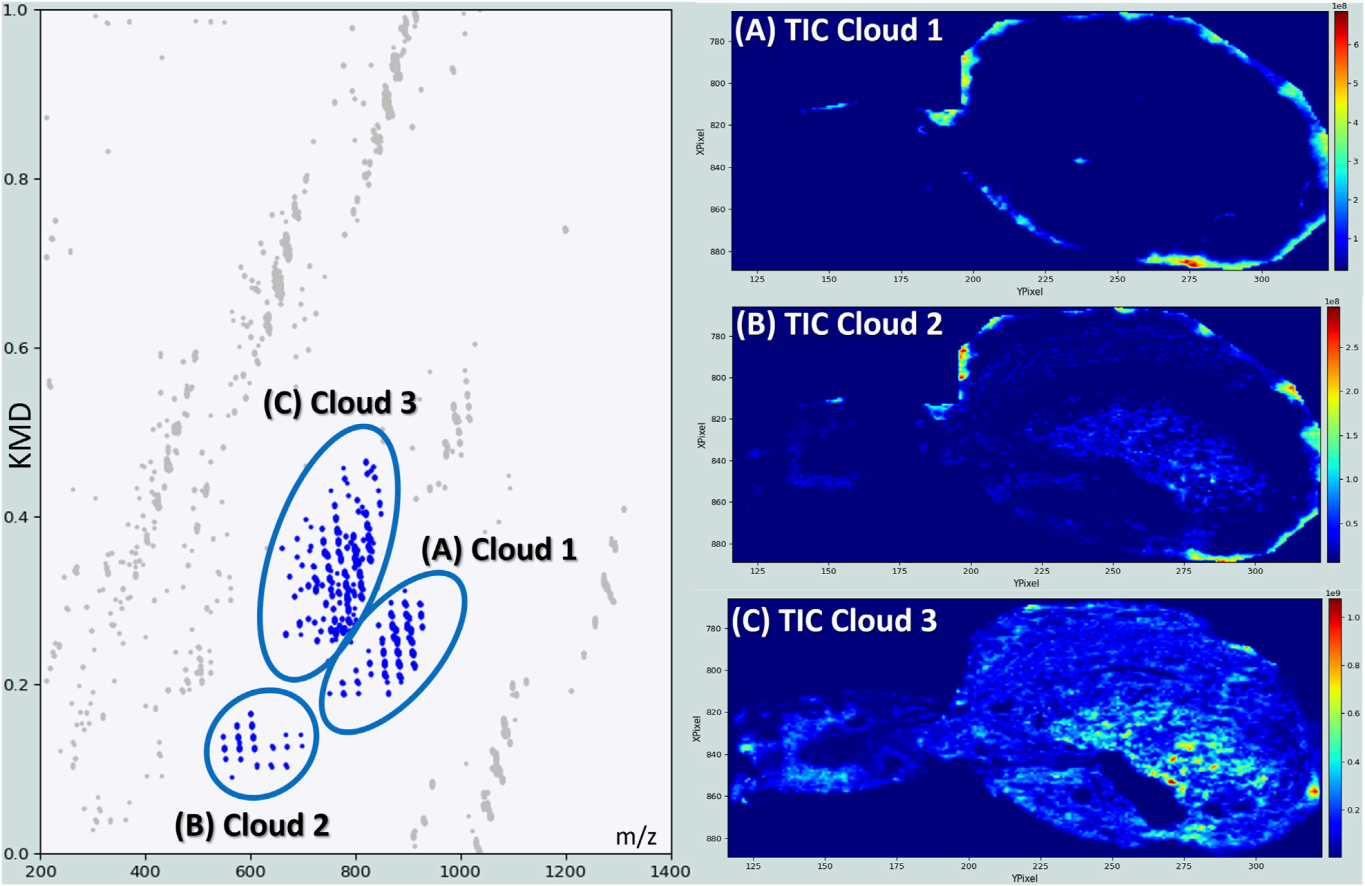
*Kendrick Mass Defect (KMD) plot and corresponding spatial distributions of ion clusters* obtained for the MSI of a female *S. nobilis.* Left: Kendrick Mass Defect (KMD) plot of detected ions from the full-body mass spectrometry imaging experiment. Three distinct clusters of biologically relevant ions (Clouds 1–3) are highlighted in blue. Right: total ion current images showing the spatial distribution of the ion clusters defined in the KMD plot. **(A)** TIC of Cloud 1, **(B)** TIC of Cloud 2, and **(C)** TIC of Cloud 3 and illustrating the accumulation of molecular species across different anatomical regions.

After a global inspection of the full KMD plot, which offers a comprehensive overview of the molecular diversity within the sample, the analysis was refined by focusing on specific “clouds”, distinct clusters of points that represent structurally related compound families. These groupings, often characterized by consistent Kendrick mass defects and coherent spatial distributions, provide a powerful means to investigate biochemical pathways, homologous series, or organ-specific lipid families with greater chemical and anatomical selectivity. Three main clouds are first investigated (Figure 4). The image associated with the compounds belonging to Cloud 1 (Figure 4A) and Cloud 2 (Figure 4B) show that they are localized in the surroundings of the spider, more precisely in the abdomen cuticle, even if anatomical structures appear lightly in the case of the Cloud 2.

Lipid identification was performed only for the most intense signals of each cloud, as the identification of the remaining compounds present in the clouds can be made at a second time, by exploiting the properties of Kendrick’s mass defects plots (series and alignments). Lipid identification was done by matching the exact masses obtained with our high-resolution FT-ICR mass spectrometer to known lipid compositions, provided by LIPIDMAPS LMSD, HMDB or Metaboscape. The exceptional mass accuracy and resolving power of FT-ICR enabled precise annotation of molecular formulas, even within complex isobaric regions. When multiple elemental compositions matched the same *m/z* value, the selection of the best candidate formula was guided by the following criteria. First, the ΔM (difference between the experimental and the theoretical masses) expressed in ppm was calculated and the composition with the lowest mass error was prioritized. Second, the polarity of ionization was used to give priority to lipids that ionize well in positive mode. Third, the molecules with high oxidation state, *i.e.*, compositions with a high number of double bonds or oxygen atoms (indicative of highly oxidized or polyunsaturated lipids) were excluded, as they were considered biologically less likely in the context of cuticular lipid composition (Drozdowski and Boguś, 2025; Gołębiowski et al., 2012; Kaczmarek et al., 2020). This strategy ensured both analytical precision and biological plausibility in the final lipid annotations. When necessary, an additional validation by manual isotopic pattern comparison was performed (see SI 4). The annotation of the most intense signals extracted for the two clouds are summarized in Table 1.

**TABLE 1:** Representative molecular assignments for ions from Cloud 1 and Cloud 2. List of selected ions detected in Clouds 1 and 2 (as defined in the Kendrick Mass Defect plot), showing their experimental mass-to-charge ratio (m/z _exp_), signal intensity, best database match, related elemental composition, ion species, theoretical mass-to-charge ratio (m/z _the_), and the mass error (ΔM) in parts per million (ppm). Cloud 1 is predominantly composed of DGs and TGs, while Cloud 2 is mainly associated with MGs or oxidized DGs. These lipides contribute to the spider protection against desiccation and may participate in chemical communication with her environment.

Cloud	m/z exp	Intensity	Best match	Ion elementary composition	Ion	m/z the	ΔM (ppm)
1	851.7103	5.03E+09	TG 50:3	C53 H96 O6 Na	[M + Na]^+^	851.7099	0.5
1	853.7261	6.23E+09	TG 50:2	C53 H98 O6 Na	[M + Na]^+^	853.7256	0.6
1	867.6846	5.37E+09	DG 53:9	C56 H92 O5 Na	[M + Na]^+^	867.6837	1.0
1	869.7000	7.87E+09	DG 53:8	C56 H94 O5 Na	[M + Na]^+^	869.6993	0.8
1	871.7158	2.60E+09	TG 50:1	C53 H100 O6 K	[M + K]^+^	871.7151	0.8
1	879.7418	8.73E+09	TG 52:3	C55 H100 O6 Na	[M + Na]^+^	879.7412	0.7
1	881.7584	7.84E+09	TG 52:2	C55 H102 O6 Na	[M + Na]^+^	881.7569	1.7
1	893.6999	2.57E+09	TG 52:4	C55 H98 O6 K	[M + K]^+^	893.6995	0.4
1	895.7166	7.62E+09	TG 52:3	C55 H100 O6 K	[M + K]^+^	895.7152	1.6
1	897.7317	6.34E+09	TG 52:2	C55 H102 O6 K	[M + K]^+^	897.7308	1.0
1	907.7737	2.61E+09	TG 54:3	C57 H104 O6 Na	[M + Na]^+^	907.7725	1.3
2	549.4883	3.00E+09	MG 30:0 or DG O-30:0	C33 H66 O4 Na	[M + Na]^+^	549.4853	5.5
2	551.5038	1.52E+09	MG 32:2 or DG O-32:2	C35 H67 O4	[M + H]^+^	551.5034	0.7
2	573.4880	2.07E+09	MG 32:2 or DG O-32:2	C35 H66 O4 Na	[M + Na]^+^	573.4853	4.7
2	575.5039	1.53E+09	MG 32:1 or DG O-32:1	C35 H68 O4 Na	[M + Na]^+^	575.5010	5.0
2	577.5187	2.87E+10	MG 32:0 or DG O-32:0	C35 H70 O4 Na	[M + Na]^+^	577.5166	3.6
2	601.5189	7.51E+09	MG 34:2 or DG O-34:2	C37 H70 O4 Na	[M + Na]^+^	601.5166	3.8
2	603.5351	1.54E+10	MG 34:1 or DG O-34:1	C37 H72 O4 Na	[M + Na]^+^	603.5323	4.6
2	605.5510	3.53E+09	MG 34:0 or DG O-34:0	C37 H74 O4 Na	[M + Na]^+^	605.5479	5.1

As shown in Figure 4, most of the lipids identified in Cloud 1 and 2 are respectively triacylglycerides (TGs). The detection of such lipids in the cuticle of *Steatoda* spiders can be rationalized by their role in waterproofing, barrier function, and possibly chemical communication. Indeed, like insects, spiders must prevent desiccation, especially given their small size and high surface-area-to-volume ratio and their cuticle layer is coated with a lipid-rich surface that minimizes water loss (Trabalon and Garcia, 2021; Wang et al., 2016). TGs are particularly hydrophobic, excellent for waterproofing and may be secreted from specialized epidermal spider glands. It is important to note that monoacylglycerides (MGs) and diacylglycerides (DGs), detected as well, may additionally result from enzymatic hydrolysis of triacylglycerides (TGs) by lipases within the cuticle or hemolymph, in the context of biosynthetic turnover. Analyzing these two groups by horizontal or vertical alignment does not change their localization, suggesting their ubiquitous presence in the cuticle, even if TG 48:2 (*m/z* 825.6943) and TG 48:1 (*m/z* 827.7099) look much intense in the spinneret’s region (see Supplementary Figure S5). Horizontal alignments reveal homologous series of compounds differing by repeating units (e.g., CH_2_), allowing the identification of lipid families with consistent structural backbones such as for example, TG 50:3, TG 52:3 and TG 54:3. Meanwhile, vertical alignments highlight nearly isobaric species, differing in their elemental composition due to variation in degrees of unsaturation or oxidation like TG 50:1, TG 50:2 and TG 50:3. Interestingly, the presence of lipids on spider cuticle has also been associated to potential antimicrobial properties and communication pheromones that could be exploited to manage, for example, spider colonization (Chajduk et al., 2024).

**FIGURE 5:**
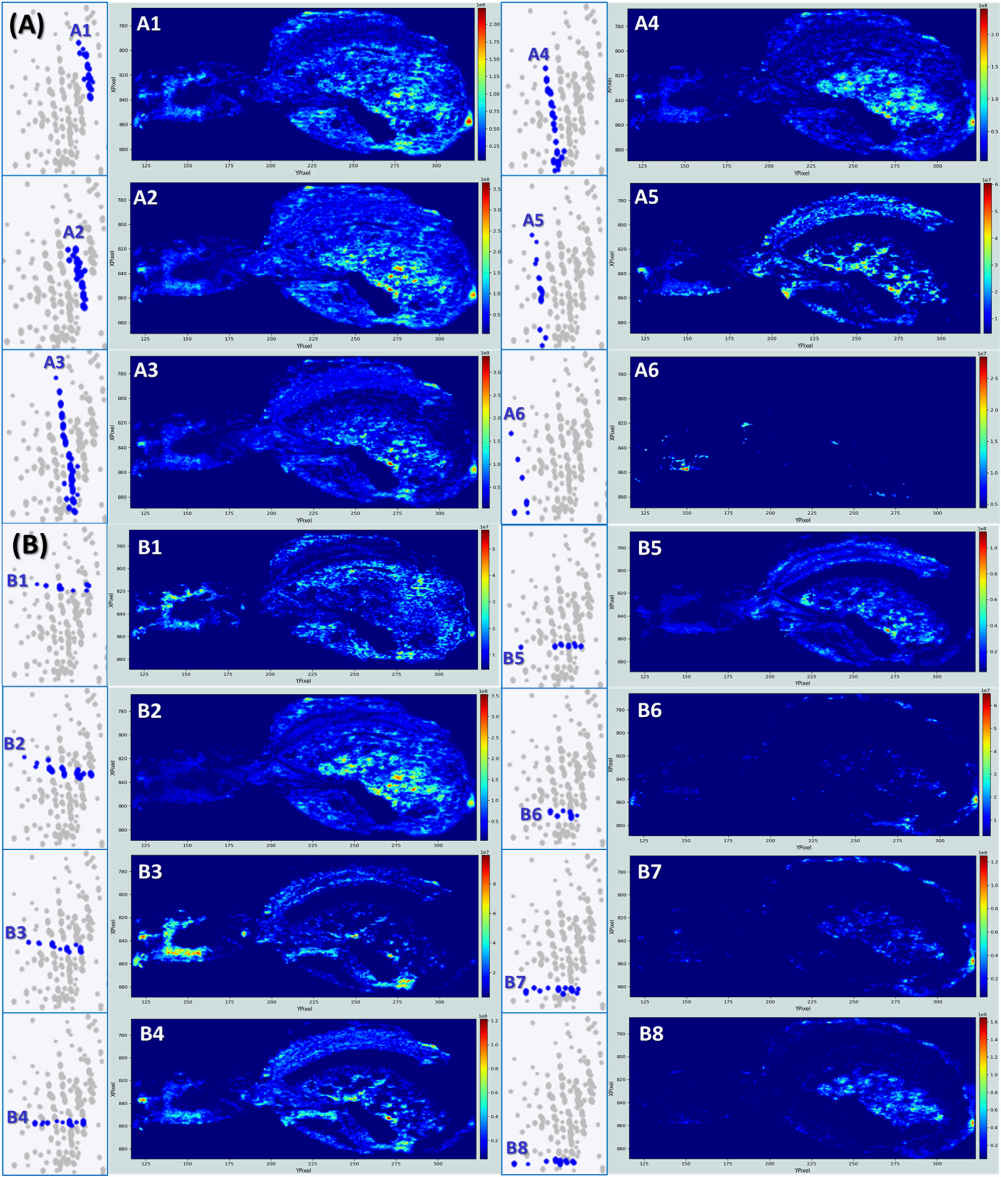
Kendrick’s mass defect plots analyzed by both vertical groupings and horizontally aligned series to reveal additional distribution of lipids in the spider tissues. **(A)** Series corresponding to the vertical distribution of ions, indicative of different functional groups or degrees of unsaturation within a lipid class. **(B)** Series corresponding to the horizontal distribution of ions, reflecting homologous series with repeating structural units (e.g., CH_2_).

Concerning Cloud 3, the most populated, the analysis is less straightforward as many compounds are not co-localized. To enhance the structural interpretation of this complex KMD space, the analysis through both vertical groupings (Figure 5A) and horizontally aligned series (Figure 5B) is proposed. This dual approach refines molecular annotation by distinguishing true biochemical series and supports structure filtering, especially in this untargeted lipidomics workflow. The annotations for the most intense signals for each strategy are summarized in Table 2.

**TABLE 2:** Detailed molecular assignments for ion series within Cloud 3. Table showing the experimental m/z values, signal intensities, best tentative matches, associated elemental compositions, detected ion species, theoretical m/z values, and mass errors (ΔM in ppm) for two main ion series (A and B) identified within Cloud 3 from the Kendrick Mass Defect analysis. *Ax* series design the vertical groupings, whereas *By* series indicate the horizontally aligned families.

Cloud	Series	m/z exp	Intensity	Best match	Ion elementary Composition	Ion	m/z the	ΔM (ppm)
3	A1	820.5254	6.23E+09	PC (36:4) or PE (39:4)	C44 H80 N O8 P K	[M + K]^+^	820.5253	0.1
3	A1	822.5398	3.59E+10	PC (36:3) or PE (39:3)	C44 H82 N O8 P K	[M + K]^+^	822.5410	1.5
3	A1	824.5559	9.95E+09	PC(36:2) or PE (39:2)	C44 H84 N O8 P K	[M + K]^+^	824.5566	0.8
3	A1	826.5722	1.01E+10	PC (36:1) or PE (39:1)	C44 H86 N O8 P K	[M + K]^+^	826.5723	0.1
3	A2	796.5251	5.01E+10	PC (34:2) or PE (37:2)	C42 H80 N O8 P K	[M + K]^+^	796.5253	0.3
3	A2	798.5416	7.73E+10	PC (34:1) or PE (37:1)	C42 H82 N O8 P K	[M + K]^+^	798.5410	0.8
3	A2	806.5667	1.53E+10	PC (36:3) or PE(39:3)	C44 H82 N O8 P Na	[M + Na]^+^	806.5670	0.4
3	A2	808.5825	1.22E+10	PC (36:2) or PE (39:2)	C44 H84 N O8 P Na	[M + Na]^+^	808.5827	0.2
3	A3	763.4667	1.18E+10	PA (38:4)	C41 H73 O8 P K	[M + K]^+^	763.4675	1.0
3	A3	765.4824	1.52E+10	PA (38:3)	C41 H75 O8 P K	[M + K]^+^	765.4831	0.9
3	A3	770.5099	3.45E+09	PC (32:1) or PE (35:1)	C40 H78 N O8 P K	[M + K]^+^	770.5097	0.3
3	A3	780.5515	2.53E+10	PC (34:2) or PE (37:2)	C42 H80 N O8 P Na	[M + Na]^+^	780.5514	0.1
3	A3	782.5678	3.83E+10	PC (34:1) or PE(37:1)	C42 H82 N O8 P Na	[M + Na]^+^	782.5670	1.0
3	A3	786.6008	4.66E+09	PC (36:2) or PE (39:2)	C44 H84 N O8 P	[M + H]^+^	786.6007	0.1
3	A4	737.4516	1.98E+10	PA (36:3)	C39 H71 O8 P K	[M + K]^+^	737.4518	0.3
3	A4	739.4676	3.21E+10	PA (36:2)	C39 H73 O8 P K	[M + K]^+^	739.4675	0.1
3	A4	747.4930	9.13E+09	PA (38:4)	C41 H73 O8 P Na	[M + Na]^+^	747.4935	0.7
3	A4	749.5087	5.76E+09	PA (38:3)	C41 H75 O8 P Na	[M + Na]^+^	749.5092	0.7
3	A4	758.5695	7.66E+09	PC (34:2) or PE (37:2)	C42 H80 N O8 P	[M + H]^+^	758.5694	0.1
3	A4	760.5842	2.45E+10	PC (34:1) or PE (37:1)	C42 H82 N O8 P	[M + H]^+^	760.5851	1.2
3	A5	721.4773	8.68E+09	PA (36:3)	C39 H71 O8 P Na	[M + Na]^+^	721.4779	0.8
3	A5	723.4932	1.98E+10	PA (36:2)	C39 H73 O8 P Na	[M + Na]^+^	723.4935	0.4
3	A5	724.4974	1.42E+09	PA (36:2) - first isotope	12C38 13C H73 O8 P Na	[M + Na]^+^	724.4970	0.6
3	A6	666.4834	3.28E+08	?	?	-	-	>10
3	A6	682.4575	1.46E+08	?	?	-	-	>19
3	A6	692.4991	5.40E+08	?	?	-	-	> 10
3	B1	763.4667	1.18E+10	PA (38:4)	C41 H73 O8 P K	[M + K]^+^	763.4675	1.0
3	B1	794.5095	7.04E+09	PC (34:3) or PE (37:3)	C42 H78 N O8 P K	[M + K]^+^	794.5097	0.3
3	B1	822.5398	3.59E+10	PC (36:3) or PE (39:3)	C44 H82 N O8 P K	[M + K]^+^	822.5410	1.5
3	B2	739.4676	3.73E+10	PA (36:2)	C39 H73 O8 P K	[M + K]^+^	739.4675	0.1
3	B2	740.4702	1.06E+10	PA (36:2) - first isotope	12C38 13C H73 O8 P K	[M + K]^+^	740.4709	0.9
3	B2	767.4999	3.02E+09	PA(38:2)	C41 H77 O8 P K	[M + K]^+^	767.4988	1.4
3	B2	770.5099	3.45E+09	PC(32:1) or PE 35:1)	C40 H78 N O8 P K	[M + K]^+^	770.5097	0.3
3	B2	798.5405	7.73E+10	PC (34:1) or PE 37:1)	C42 H82 N O8 P K	[M + K]^+^	798.5410	0.6
3	B2	804.5505	7.73E+10	PC (36:4) or PE 39:4)	C44 H80 N O8 P Na	[M + Na]^+^	804.5514	1.1
3	B3	747.4930	9.13E+09	PA (38:4)	C41 H73 O8 P Na	[M + Na]^+^	747.4935	0.7
3	B3	749.5087	5.75E+09	PA (38:3)	C41 H75 O8 P Na	[M + Na]^+^	749.5092	0.7
3	B3	778.5357	1.76E+10	PC (34:3) or PE (37:3)	C42 H78 N O8 P Na	[M + Na]^+^	778.5357	0.0
3	B3	806.5668	1.53E+10	PC (36:3) or PE (39:3)	C44 H82 N O8 P Na	[M + Na]^+^	806.5670	0.2
3	B3	808.5823	8.95E+09	PC (36:2) or PE (39:2)	C44 H84 N O8 P Na	[M + Na]^+^	808.5827	0.5
3	B4	780.5515	2.53E+10	PC (34:2)or PE (37:2)	C42 H82 N O8 P Na	[M + Na]^+^	780.5514	0.1
3	B4	808.5825	1.22E+10	PC (36:2) or PE (39:2)	C44 H84 N O8 P Na	[M + Na]^+^	808.5827	0.2
3	B5	767.5464	5.83E+08	SM (36:2; O2)	C41 H81 N2 O6 P K	[M + K]^+^	767.5464	0.0
3	B5	782.5663	3.83E+10	PC (34:1) or PE (37:1)	C42 H82 N O8 P Na	[M + Na]^+^	782.5670	0.9
3	B5	810.5988	1.77E+09	PC (36:1) or PE (39:1)	C44 H86 N O8 P Na	[M + Na]^+^	810.5983	0.6
3	B6	756.5548	1.77E+08	PC (34:3) or PE (37:3)	C42 H78 N O8 P	[M + H]^+^	756.5538	1.3
3	B6	784.5856	4.24E+09	PC (36:3) or PE (39:3)	C44 H82 N O8 P	[M + H]^+^	784.5851	0.6
3	B7	692.4991	5.40E+08	?	?	-	-	>> 10
3	B7	758.5696	7.66E+09	PC (34:2) or PE (37:2)	C42 H80 N O8 P	[M + H]^+^	758.5694	0.3
3	B7	786.6008	4.47E+09	PC (36:2) or PE (39:2)	C44 H84 N O8 P	[M + H]^+^	786.6007	0.1
3	B8	760.5842	2.45E+10	PC (34:1) or PE (37:1)	C42 H82 N O8 P	[M + H]^+^	760.5851	1.2
3	B8	774.6004	2.33E+09	PC (35:1) or PE (38:1)	C43 H84 N O8 P	[M + H]^+^	774.6007	0.4
3	B8	788.6159	7.59E+08	PC (36:1) or PE (39:1)	C44 H86 N O8 P	[M + H]^+^	788.6164	0.6

### Vertical groups A1, A2 and A3

Vertical groups A1 and A2, are mainly made of phosphatidylcholine PC(34:X) and PC(36:Y), and(/or) their phosphatidylethanolamine isomers PE(37:X) and PE(39:Y). Distinguishing any isomer couple PC(X:Y) and PE(X+3:Y) would necessitate for instance additional LC-MS, as PCs are generally more polar than PEs and therefore elute differently (Triebl et al., 2017). It can also be done using MS/MS experiments as in positive ion mode, PCs are typically identified by a distinctive fragment ion at m/z 184, corresponding to the phosphocholine headgroup, whereas PEs often exhibit a neutral loss of 141 Da, associated with the loss of the phosphoethanolamine moiety (Baker et al., 2014). As MSI does not provide extensive fragmentation data, definitive distinction between PCs and PEs remains difficult at this stage. Lipids from A1 and A2 share globally the same distribution within the spider, present especially in the digestive tubules and the ovaries which reflect their roles as major membrane phospholipids (Ma et al., 2024). Their detection in the rectum/anus area may relate to membrane turnover or lipid-rich excretion pathways.

### Vertical groups A4 and A5

Other interesting distributions are the A4 and the A5 where lipids are essentially detected in the ovaries. The signal mainly results from the presence of long-chain phosphatidic acids like PA (36:2), PA (36:3), PA (38:3), and PA (38:4). This makes sense as PAs are the building-block precursors to most phospholipids. In developing ovarian tissue, membrane synthesis and remodeling are very active, which requires the constant synthesis of these complex phospholipids. Moreover, lipids constitute around 30% of the dry weight of insect oocytes, participating in the embryo’s development as the main source of energy (Arrese and Soulages, 2010). Intriguingly, PA (36:2) and PA (36:3) are detected on the ovary’s shells under their potassium adducts [M + K]^+^ (A4), whereas the same lipids are detected under their sodium adducts [M + Na]^+^ in the matrix surrounding the ovaries (A5). This may suggest a different balance between sodium and potassium between the follicular fluid/membrane and the surroundings. Although direct studies on potassium and sodium concentrations in spider ovaries have not been performed yet, a study on *Drosophila* ovarian follicles tends to confirm this idea, affirming the crucial role of potassium transport in follicles during oocyte development (Bohrmann, 1991).

### Vertical group A6

Group A6 is associated with molecules predominantly detected in the cephalothorax. While their identities remain unknown, their restricted localization raises the possibility that they may contribute to neural processes within the spider’s central nervous system.

### Horizontal groups B1 and B3

When looking at the horizontally aligned lipids, a particularly impressive distribution is found in B1 (Figure 5). The image shows an intense detection of lipids in the spinnerets area, in the nervous central system, as the brain and the eyes are clearly highlighted, and in a zone that could be the venom glands and the chelicerae, or more likely the stomach and the mouth area. The contrast is even more marked for the B3 distribution. B1 and B3 distributions are mainly due to PC (34:X) and PC (36:X) and/or PE (37:Y) or PE (39:Y), and PA 38:4. More precisely, PA (38:3) and PA (38:4) are strongly detected in the brain compared to the other species (see Supplementary Figure S6). The prominent presence of these phosphatidic acids in the brain and central nervous system of spiders can be explained by their recognized roles in neural tissues across various species. PA (38:3) has been for example, detected in white matter areas of rat brains (Martínez-Gardeazabal et al., 2017). As already discussed, PAs serve as precursors for the biosynthesis of major membrane phospholipids, contributing to neuronal membrane integrity and, here, synaptic function (Ammar et al., 2014). Additionally, PAs act as a signaling lipid, regulating intracellular pathways pertinent to neurosecretion, membrane trafficking, and cytoskeletal dynamics processes integral to neural activity (Ammar et al., 2014). While no specific studies on spiders can be found to date, the conservation of lipid-mediated mechanisms in neural tissues suggests that PA 38:3 and 38:4 plays similar roles in arachnid neurobiology. Phosphatidic acids appear intense in the spider eyes as well. This is also in line with studies on *Drosophila* which have demonstrated, for instance, the importance of PAs in membrane dynamics essential for proper photoreceptor genesis and function (Raghu et al., 2009).

### Horizontal group B4

The distribution B4 is dominated by the signal detected at *m/z* 780.5515 and associated with possibly PC (34:2) and/or PE (37:2). They are intensively detected in the heart and in the eyes of the spider, which is in accordance with the known physiological roles of these phospholipids in various species (Lim et al., 2011; Stark et al., 1993; Zhao and Wang, 2020). The heart of the spider, even simple, is a highly active muscle metabolically and the importance of PCs in the cardiovascular system has been demonstrated several times. For example, a higher consumption of PCs increases mortality from cardiovascular diseases (Zheng et al., 2016). PCs and PEs additionally play an important role in the eyes, especially in the photoreceptor membrane structure, essential for ocular function. For example, PE forms a Schiff base conjugate with trans-retinal (Vitamin A) to form the N-RET-PE molecule, crucial for the proper functioning of the visual process and pigment photogeneration(Xu et al., 2023).

**FIGURE 6:**
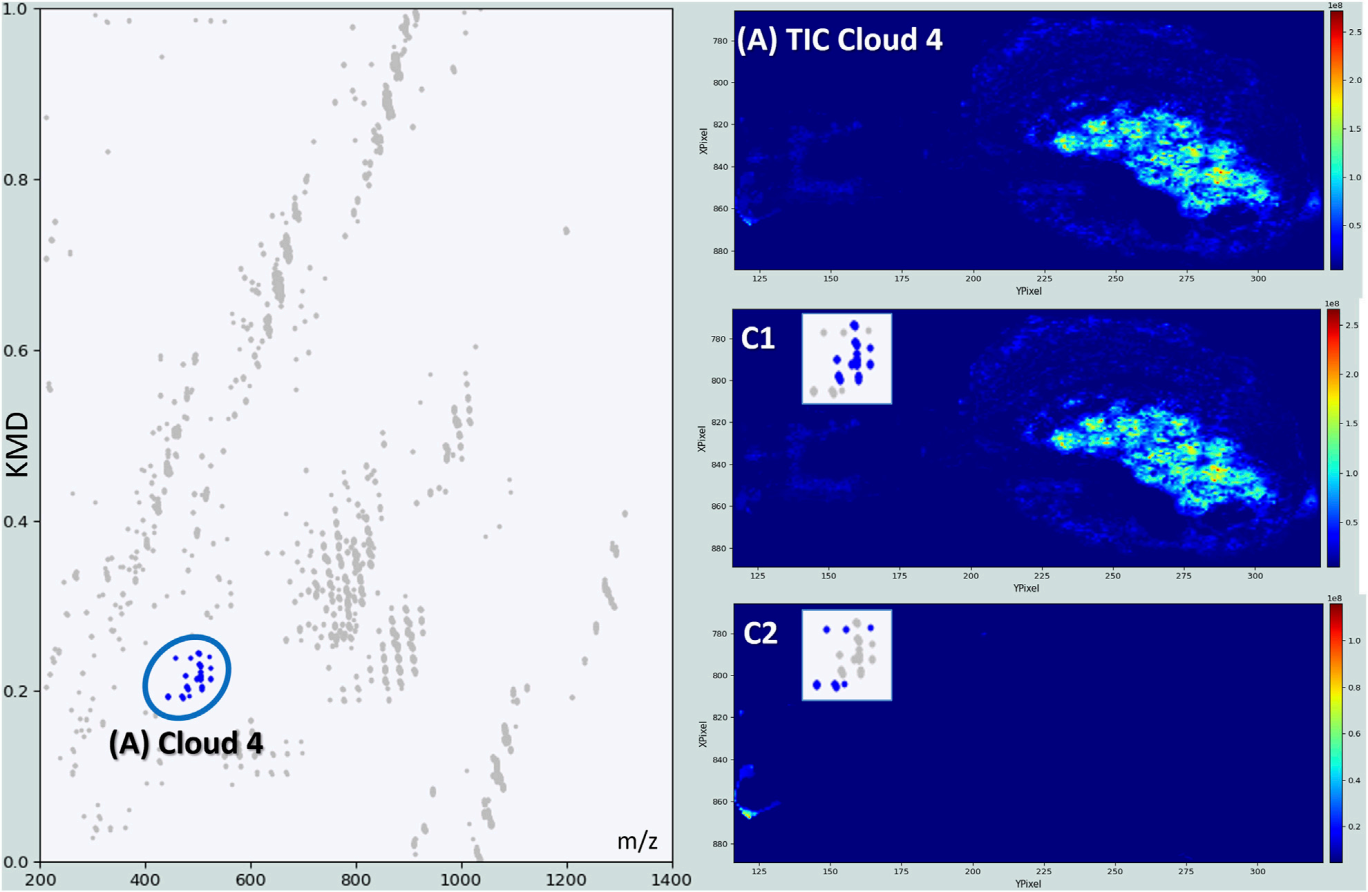
Kendrick’s mass defects plot obtained for the MSI of a female *S. nobilis*. Displayed images are related to the Total ion current (Top), and to two secondary groups C1, built from ions localized in the ovaries, and C2 related to species detected in the fang area of the spider.

Another Cloud of dots reveals additional information about the *S. nobilis* lipidome (Figure 6). These ions are visibly mainly localized in the ovaries/eggs of the spider. However, looking in much detail, they can be classified according to two groups. The first one (Figure 6–C1) concerns lipids strongly detected in the ovaries. These molecules correspond to lysophosphatidylcholines (LPCs) and lysophosphatidylethanolamines (LPEs), phospholipids missing one of their fatty acid chains usually removed by a lipase (Table 3).

**TABLE 3:** Detailed molecular assignments for ion series within Cloud 4. Table showing the experimental m/z values, signal intensities, best tentative matches, associated elemental compositions, detected ion species, theoretical m/z values, and mass errors (ΔM in ppm) for the ions identified within Cloud 4 from the Kendrick Mass Defect analysis. Lysophospholipids are precisely located in the spider ovaries, whereas sterol molecules are most intensively detected in the chelicerae.

Cloud	Series	m/z exp	Intensity	Best match	Ion elementary composition	Ion	m/z the	ΔM (ppm)
4	C1	476.3133	6.29E+08	LPC (O-14:0)	C22 H48 N O6 P Na	[M + Na]^+^	476.3111	4.6
4	C1	478.3289	1.81E+10	LPE (O-19:2)	C24 H48 N O6 P	[M + H]^+^	478.3292	0.6
4	C1	500.3135	6.19E+07	LPE (O-21:2)	C26 H52 N O6 P	[M + Na]^+^	500.3111	4.8
4	C1	502.3289	2.93E+10	LPC (O-16:1)	C24 H50 N O6 P Na	[M + Na]^+^	502.3268	4.2
4	C1	504.3444	8.60E+10	LPC (O-16:0)	C24 H52 N O6 P Na	[M + Na]^+^	504.3424	4.0
4	C1	506.3604	4.11E+09	LPC (O-18:2)	C26 H52 N O6 P	[M + H]^+^	506.3605	0.2
4	C2	441.2978	5.40E+08	ST 26:2; O4	C26 H42 O4 Na	[M + Na]^+^	441.2975	0.7
4	C2	469.3293	7.68E+08	ST 28:2; O4	C28 H46 O4 Na	[M + Na]^+^	469.3288	1.1

The detection of LPCs and LPEs in the ovaries and eggs of spiders can be attributed to their crucial roles in lipid metabolism and membrane dynamics during oocyte development and embryogenesis (Steinhauer et al., 2009). Although LPEs are less studied, LPCs have been identified as main lipid components of lipovitellins isolated from the eggs of the wolf spider *Schizocosa malitiosa* (Laino et al., 2013). Lipovitellins are major yolk proteins that serve as energy sources during embryonic development in oviparous species. The detection of LPCs in these lipovitellins indicates their significant role in the reproductive biology of spiders, likely contributing to membrane dynamics and lipid metabolism during oogenesis and embryogenesis.

The second series is composed of molecules mostly localized in the chelicerae area (Figure 6–C2). Chelicerae are kind of cuticular extensions involved in feeding and mechanical delivery of the venom into the prey. The signals are strongly dominated by molecules belonging much probably to the sterol family, never described in this spider organ. Considering however, that insect cuticles contain sterols (Wrońska et al., 2023) and that the mandibular glands of *Apis mellifera* secrete sterols as well (Buttstedt et al., 2023), detecting such molecules in the biting apparatus of *S. nobilis* aligns very well with its cuticular structure and its secretory function.

### KMD plots for single metabolite analysis

**FIGURE 7:**
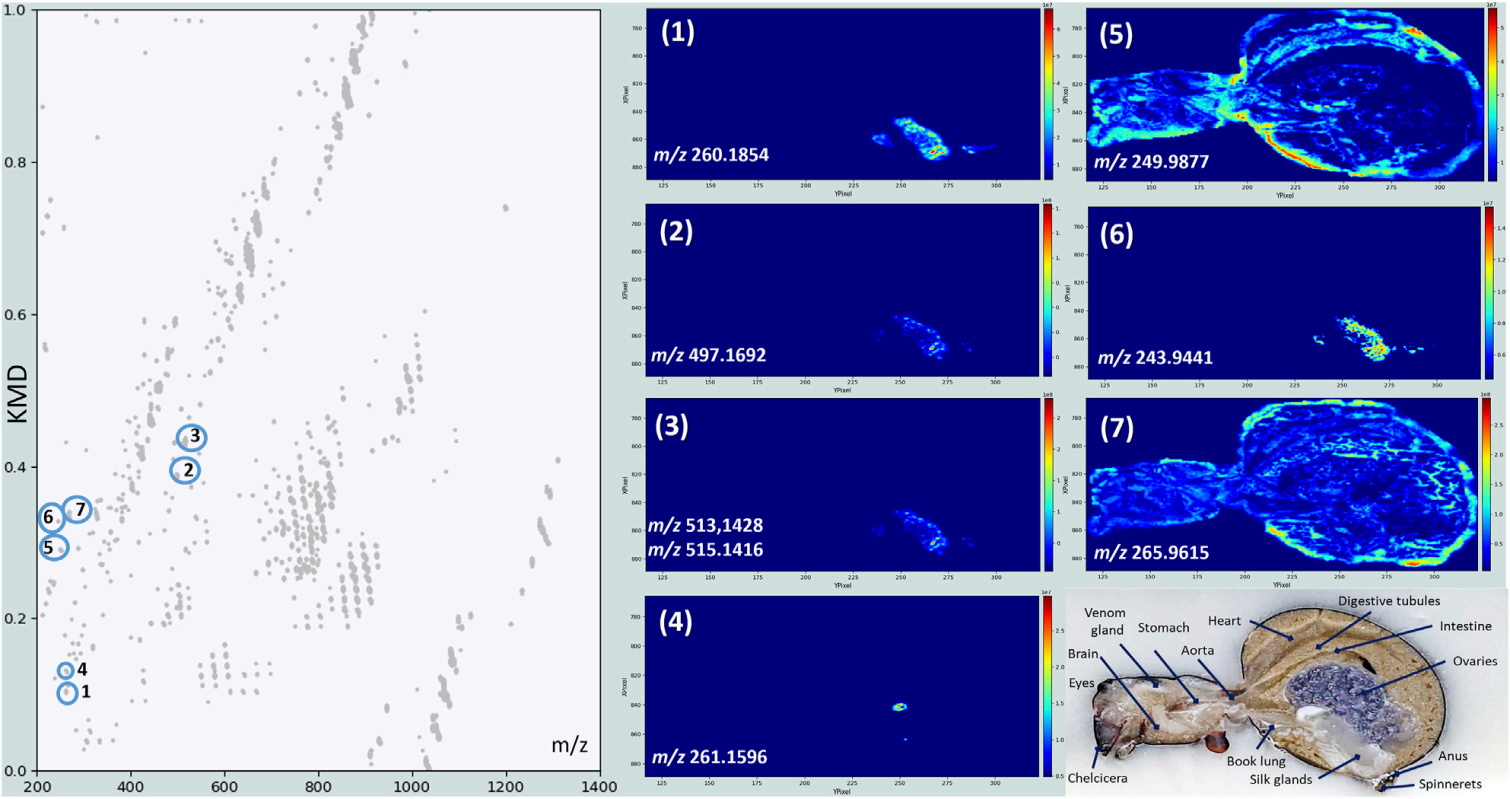
Kendrick’s mass defects plot obtained for the MSI of a female *S. nobilis*. Displayed images correspond to the individual metabolites labelled in the plot. The silk glands (1–3 and 6), the oviduct (4), the pericardium (5) and the digestive tubules (7) are very well detected.

In addition to global lipid class analysis, KMD plots can also be used to follow individual metabolites, which do not belong to lipid classes. When using a CH_2_ base, compounds that are not aligned horizontally do not belong, by definition, to repeating CH_2_-based compound classes, making them easily detectable within complex datasets. Figure 7 is dedicated to these compounds and shows atypical distributions for eight of them. Metabolites 1, 2, 3 and 6 display the same distribution and are detected with high intensity in particular organs, identified as the silk glands. One of these metabolites is accurately identified as hexanoylcarnitine (*m/z* 260.1854). Carnitines play a pivotal role in mitochondrial fatty acid transport and β-oxidation, allowing cells to generate ATP from fatty acids (Virmani and Cirulli, 2022). Their detection by MALDI-MSI in the silk glands of *S. nobilis* appears to be biologically coherent, as silk production is a highly energy-demanding process requiring sustained ATP supply (Xu et al., 2022). The presence of hexanoylcarnitine specifically suggests the mobilization of medium-chain fatty acids, indicating that these are actively oxidized to meet the energetic demands of silk protein synthesis and secretion. While direct studies on carnitine’s function in spider silk glands do not exist, the established importance of carnitine in energy-demanding processes in insects implies a similar role in arachnids (Childress et al., 1967).

The metabolite responsible for the signal 4 could match the cyclic dipeptide Leu-Phe (c-Leu-Phe), as the mass corresponds very well (*m/z* 261.1596, ΔM = 0.4 ppm). This metabolite might be endogenous, as a product of peptide and protein degradation, or exogenous, originating from microbial or environmental sources. It is located in a very specific zone, just between the ovaries/eggs and the silk glands. The identification of this organ is not straightforward as only a few histological slices of spiders are documented. Our hypothesis is that this organ may be the oviduct of the spider, from where the eggs are expulsed before being wrapped in a silk cocoon. Metabolite 5 is intensely detected in the close surrounding of the heart, the pericardium. As the spider heart supports hemolymph circulation, pericardial cells need readily available energy (Meyer et al., 2024). The nature of the metabolite would probably support the local energy requirements near the heart or reflect the metabolic buffering role of pericardial cells in processing circulating lipid and lipid derivatives.

Metabolite 7 (*m/z* 265.9615) displays a very interesting distribution. Its signal is more intense in the interstices between the digestive tubules, giving birth to additional histological structures. Its nature is, however, still unidentified, not only because spider metabolome information is scarce, but also because this area is involved in exchange between the digestive epithelium and hemolymph (allowing for instance the nutrient uptake), and a large variety of molecules, possessing various physico-chemical properties, can therefore be present in this place.

## Discussion

This study delivers the first organ-specific, spatially resolved lipidomic and metabolomic map of a spider, here the noble false widow spider, *S. nobilis*. It has been achieved through an integrative workflow combining high-resolution Matrix-Assisted Laser Desorption/Ionization Mass Spectrometry Imaging (MALDI-MSI) with Kendrick Mass Defect (KMD) analysis. This powerful analytical framework enabled us to visualize and classify molecular species with suborgan selectivity, offering new insights into the internal chemical anatomy of an arachnid, a group of animals for which molecular-level spatial data remains virtually poorly if not nonexistent. KMD plots were powerful not only for lipid class separation but also for the detection of unusual or outlier metabolites. These plots allowed us to group chemically homologous series and to detect structural analogs even in the absence of prior annotation. The dual capability of KMD, both as a classifier and a screening tool, was crucial in this exploratory context, where spectral libraries are sparse and manual validation is limited. Importantly, the combination of KMD with FT-ICR’s sub-ppm mass accuracy allowed for high-confidence elemental composition predictions, a major advantage for metobolomic studies in non-model species. Globally, given the significant biological and ecological roles that lipids and small metabolites play in invertebrate physiology, this work addresses a critical knowledge gap and introduces a versatile, transferable method for studying the functional biochemistry of non-model organisms.

The preservation of anatomical integrity through optimized cryosectioning and gelatin embedding protocols allowed us to retain spatial context during mass spectrometry analysis. This was essential for mapping hundreds of compounds across the complex internal landscape of the spider body, where organs are compact and highly specialized. The resulting molecular maps revealed organ-specific lipid signatures that correspond to physiological functions known or hypothesized from related taxa. In the outer body structures, the cuticle and spinnerets as well, triacylglycerides were dominant. These hydrophobic compounds are likely involved in maintaining water balance and structural integrity under desiccating terrestrial conditions. Their presence near silk-producing spinnerets also suggests a potential role in facilitating silk extrusion through lubrication or structural stabilization of the silk proteins. Mono- and diacylglycerides, detected broadly in these same regions, may represent lipid turnover intermediates, possibly derived from energy mobilization or membrane remodeling in response to environmental or mechanical stimuli. The internal tissues, particularly those associated with reproduction, revealed a markedly different lipid profile. Ovarian regions showed high concentrations of phosphatidic acids (PAs), which are key intermediates in the biosynthesis of complex phospholipids. These molecules are essential for rapid membrane formation during oogenesis, and their presence supports the hypothesis of active lipid remodeling and trafficking within the developing oocytes. The spatial detection of different adduct forms of these molecules, such as [M + Na]^+^ and [M + K]^+^, in distinct microregions of the ovaries suggests that ionic microenvironments may influence lipid dynamics and compartmentalization, an aspect rarely captured in conventional metabolomic studies. Additional lipid species such as lysophosphatidylcholines (LPCs) and lysophosphatidylethanolamines (LPEs) were also localized in the reproductive system, including egg masses. These lysolipids are known to result from phospholipase-mediated cleavage and are implicated in signaling pathways and membrane restructuring. Their detection in reproductive tissues aligns with their previously proposed function in nutrient provisioning and yolk formation in spider embryos, as supported by earlier findings, in *Schizocosa malitiosa* for instance. In the central nervous system, comprising the brain and optic centers, phosphatidylcholines (PCs) and phosphatidylethanolamines (PEs) were intensively detected. These lipids are foundational components of synaptic and axonal membranes, and their abundance here is consistent with high turnover and maintenance demands required by sensory processing and neural communication. Moreover, the co-localization of phosphatidic acids in these regions reinforces their putative involvement in synaptic vesicle formation, signaling, and other neurophysiological roles. One of the most novel findings of this study was the accumulation of hexanoylcarnitine in the silk glands. Carnitines are involved in transporting fatty acids into mitochondria for β-oxidation, and their enrichment in silk-producing tissues strongly suggests a high energetic demand associated with silk biosynthesis. While the mechanical and biochemical cost of silk production in spiders is well known, this is the first time that direct molecular evidence of energy metabolism has been spatially mapped to the silk glands themselves. Another distinctive pattern emerged in the chelicerae and fang region, where sterol-like compounds were highly concentrated. Sterols are critical for membrane fluidity and cuticle structure, and their presence in these mechanically active venom-associated regions suggests a dual role in structural reinforcement and possibly in venom solubilization or delivery. Comparable roles have been observed in insect mandibular glands, hinting at a conserved lipid function across arthropod lineages. While many metabolites could be assigned tentative identities based on exact mass and localization, several compounds remained unannotated yet showed highly specific spatial distributions. For instance, a feature consistent with the cyclic dipeptide Leu-Phe was localized to the oviduct area, possibly reflecting proteolytic activity or microbiota interactions. Other unidentified metabolites clustered around the heart and pericardial cavity may be involved in lipid-derived signaling or energy buffering in the hemolymph system, while small molecules detected in digestive tubules likely participate in nutrient processing and absorption.

Despite these achievements, the lack of tandem MS (MS/MS) data for many ions remains a limiting factor for unambiguous structural identification. Moreover, while the instrument used in this study is capable of delivering mass accuracies typically better than 1 ppm, we observed that some detected features exhibited higher mass errors, occasionally exceeding 4 ppm. These deviations can be due to space-charge effects, which are known to impact mass accuracy in high-abundance or spatially complex samples, such as full-body sections or to low abundances of some molecules. Furthermore, due to the limited coverage of arachnid lipidomes and metabolomes in current spectral databases, the proposed molecular annotations should be considered tentative, particularly when the associated mass error is elevated. In summary, cases where ΔM exceeds 4 ppm, annotations must be considered as hypothetical and should be interpreted with caution.

Future integration of laser microdissection followed by LC-MS/MS, or coupling to ion mobility spectrometry, would provide orthogonal validation and enhance compound resolution. Nevertheless, the anatomical selectivity and mass accuracy afforded by MALDI-MSI and KMD together form a powerful platform for generating hypothesis-driven insights even in data-poor systems like spiders.

## Conclusion

In conclusion, this study provides the first molecular atlas of the noble false widow spider, *S. nobilis*, capturing the spatial organization of lipids and metabolites across its internal organs. By combining high-resolution mass spectrometry imaging (MSI) with Kendrick mass defect (KMD) analysis, we demonstrate a robust and versatile approach for deciphering the molecular architecture of small, structurally complex organisms. This methodological framework enables the mapping of hundreds of molecular species in anatomical context, offering novel insights into spider physiology and organ-specific biochemistry.

Our findings represent a major advancement in arachnid lipidomics, revealing both conserved and unique lipid features in a terrestrial invertebrate. They fill a significant gap in comparative lipidomics and provide a valuable reference point for future investigations. Beyond spiders, this work sets the stage for broader applications of spatial lipidomics in other invertebrate systems, particularly those previously inaccessible to detailed biochemical analysis. By establishing a functional and anatomical link between molecular profiles and tissue identity, this study opens new avenues for exploring developmental biology, ecological adaptation, and evolutionary physiology across arthropods and beyond.

## Data Availability

The data presented in the study are deposited in the METASPACE repository, accession number 2025-08-19_22h18m37s under the name Spider_Steatoda_nob_female_CD_1.

## Abbreviations

CHCA, α-Cyano-4-hydroxycinnamic acid; DG, Diacylglyceride; FT-ICR, Fourier Transform Ion Cyclotron Resonance; ITO, Indium-Tin Oxide; KMD, Kendrick Mass Defect; LC-MS/MS, Liquid Chromatography-Tandem Mass Spectrometry; LPC, Lysophosphatidylcholine; LPE, Lysophosphatidylethanolamine; MG, Monoacylglyceride; MS, Mass Spectrometry; MSI, Mass Spectrometry Imaging; OCT, Optimal Cutting Temperature; PA, Phosphatidic Acid; PC, Phosphatidylcholine; PE, Phosphatidylethanolamine; ppm, Parts Per Million; ROI, Region of Interest; ST, Sterol; TG, Triacylglyceride; TIC, Total Ion Current.

## References

[B1] Abu Sammour D. Cairns J. L. Boskamp T. Marsching C. Kessler T. Ramallo Guevara C. (2023). Spatial probabilistic mapping of metabolite ensembles in mass spectrometry imaging. Nat. Commun. 14, 1823. 10.1038/s41467-023-37394-z 37005414 PMC10067847

[B2] Alseekh S. Aharoni A. Brotman Y. Contrepois K. D’Auria J. Ewald J. (2021). Mass spectrometry-based metabolomics: a guide for annotation, quantification and best reporting practices. Nat. Methods 18, 747–756. 10.1038/s41592-021-01197-1 34239102 PMC8592384

[B3] Ammar M. R. Kassas N. Bader M. F. Vitale N. (2014). Phosphatidic acid in neuronal development: a node for membrane and cytoskeleton rearrangements. Biochimie 107, 51–57. 10.1016/j.biochi.2014.07.026 25111738

[B4] Arrese E. L. Soulages J. L. (2010). Insect fat body: energy, metabolism, and regulation. Annu. Rev. Entomol. 55, 207–225. 10.1146/annurev-ento-112408-085356 19725772 PMC3075550

[B5] Baker P. R. S. Armando A. M. Campbell J. L. Quehenberger O. Dennis E. A. (2014). Three-dimensional enhanced lipidomics analysis combining UPLC, differential ion mobility spectrometry, and mass spectrometric separation strategies. J. Lipid Res. 55, 2432–2442. 10.1194/jlr.D051581 25225680 PMC4617145

[B6] Belhaj M. R. Lawler N. G. Hoffman N. J. (2021). Metabolomics and lipidomics: expanding the molecular landscape of exercise biology. Metabolites 11, 151. 10.3390/metabo11030151 33799958 PMC8001908

[B8] Bohrmann J. (1991). “ *In vitro* culture of drosophila ovarian follicles: the influence of different media on development,” in RNA synthesis, protein synthesis and potassium uptake. 10.1007/BF01705924 28305435

[B9] Burguet P. La Rocca R. Kune C. Tellatin D. Stulanovic N. Rigolet A. (2024). Exploiting differential Signal filtering (DSF) and image structure filtering (ISF) methods for untargeted mass spectrometry imaging of bacterial metabolites. J. Am. Soc. Mass Spectrom. 35, 1743–1755. 10.1021/jasms.4c00129 39007645

[B10] Buttstedt A. Pirk C. W. W. Yusuf A. A. (2023). Mandibular glands secrete 24-methylenecholesterol into honey bee (Apis mellifera) food jelly. Insect Biochem. Mol. Biol. 161, 104011. 10.1016/j.ibmb.2023.104011 37716535

[B11] Chajduk M. Tkaczuk C. Gołębiowski M. (2024). Review of the cuticular lipids of spiders (araneae). Eur. J. Entomol. 121, 73–82. 10.14411/eje.2024.011

[B12] Chen L. jun zhi Z. Zhou X. wei Xing X. yi Lv B. (2023). Integrated transcriptome and metabolome analysis reveals molecular responses of spider to single and combined high temperature and drought stress. Environ. Pollut. 317, 120763. 10.1016/j.envpol.2022.120763 36503821

[B13] Childress C. C. Sacktor B. Traynor D. R. (1967). Function of carnitine in the fatty acid oxidase-deficient insect flight muscle. J. Biol. Chem. 242, 754–760. 10.1016/s0021-9258(18)96269-1 6017744

[B14] Connor A. Zha R. H. Koffas M. (2024). Production and secretion of recombinant spider silk in Bacillus megaterium. Microb. Cell Fact. 23, 35. 10.1186/s12934-024-02304-5 38279170 PMC10821235

[B15] Conroy M. J. Andrews R. M. Andrews S. Cockayne L. Dennis E. A. Fahy E. (2024). LIPID MAPS: update to databases and tools for the lipidomics community. Nucleic Acids Res. 52, D1677–D1682. 10.1093/nar/gkad896 37855672 PMC10767878

[B16] Damm M. Vilcinskas A. Lüddecke T. (2025). Mapping the architecture of animal toxin systems by mass spectrometry imaging. Biotechnol. Adv. 81, 108548. 10.1016/j.biotechadv.2025.108548 40049423

[B17] Drozdowski M. Boguś M. I. (2025). Compartmentalization of free fatty acids in blood-feeding Tabanus bovinus females. Insects 16, 696. 10.3390/insects16070696 40725326 PMC12295098

[B18] Dugon M. M. Dunbar J. P. Afoullouss S. Schulte J. McEvoy A. English M. J. (2017). Occurrence, reproductive rate and identification of the non-native noble false widow spider Steatoda nobilis (Thorell, 1875) in Ireland. Biol. Environ. 117B, 77–89. 10.1353/bae.2017.0005

[B19] Dugon M. M. Lawton C. Sturgess D. Dunbar J. P. (2023). Predation on a pygmy shrew, Sorex minutus, by the noble false widow spider, Steatoda nobilis. Ecosphere 14, e4422. 10.1002/ecs2.4422

[B20] Dunbar J. P. Afoullouss S. Sulpice R. Dugon M. M. (2018a). Envenomation by the noble false widow spider *Steatoda nobilis* (thorell, 1875) – five new cases of steatodism from Ireland and Great Britain. Clin. Toxicol. 56, 433–435. 10.1080/15563650.2017.1393084 29069933

[B21] Dunbar J. P. Ennis C. Gandola R. Dugon M. M. (2018b). Biting off more than one can chew: first record of the non-native noble false widow spider Steatoda nobilis (thorell, 1875) feeding on the native viviparous lizard Zootoca vivipara (lichtenstein, 1823) in Ireland. Biol. Environ. Proc. R. Ir. Acad. 118B, 45. 10.3318/bioe.2018.05

[B22] Dunbar J. P. Fort A. Redureau D. Sulpice R. Dugon M. M. Quinton L. (2020a). Venomics approach reveals a high proportion of lactrodectus-like toxins in the venom of the noble falsewidow spider Steatoda nobilis. Toxins (Basel) 12, 402. 10.3390/toxins12060402 32570718 PMC7354476

[B23] Dunbar J. P. Khan N. A. Abberton C. L. Brosnan P. Murphy J. Afoullouss S. (2020b). Synanthropic spiders, including the global invasive noble false widow Steatoda nobilis, are reservoirs for medically important and antibiotic resistant bacteria. Sci. Rep. 10, 20916. 10.1038/s41598-020-77839-9 33262382 PMC7708416

[B24] Dunbar J. P. Vitkauskaite A. Lawton C. Waddams B. Dugon M. M. (2022a). Webslinger vs. dark knight first record of a false widow spider Steatoda nobilis preying on a pipistrelle bat in britain. Ecosphere 13, e3959. 10.1002/ecs2.3959

[B25] Dunbar J. P. Vitkauskaite A. O’Keeffe D. T. Fort A. Sulpice R. Dugon M. M. (2022b). Bites by the noble false widow spider *Steatoda nobilis* can induce *latrodectus* -like symptoms and vector-borne bacterial infections with implications for public health: a case series. Clin. Toxicol. 60, 59–70. 10.1080/15563650.2021.1928165 34039122

[B27] Foelix R. (2010). Biology of spiders. ed. 2010. Oxford University Press.

[B28] Gołębiowski M. Boguś M. I. Paszkiewicz M. Wieloch W. Włóka E. Stepnowski P. (2012). The composition of the cuticular and internal free fatty acids and alcohols from Lucilia sericata males and females. Lipids 47, 613–622. 10.1007/s11745-012-3662-5 22415221 PMC3357471

[B29] Hajnajafi K. Iqbal M. A. (2025). Mass-spectrometry based metabolomics: an overview of workflows, strategies, data analysis and applications. Proteome Sci. 23, 5. 10.1186/s12953-025-00241-8 40420110 PMC12105183

[B31] Hustin J. Kune C. Far J. Eppe G. Debois D. Quinton L. (2022). Differential kendrick’s plots as an innovative tool for lipidomics in complex samples: comparison of liquid chromatography and infusion-based methods to sample differential Study. J. Am. Soc. Mass Spectrom. 33, 2273–2282. 10.1021/jasms.2c00232 36378810

[B33] Kaczmarek A. Wrońska A. K. Kazek M. Boguś M. I. (2020). Metamorphosis-related changes in the free fatty acid profiles of sarcophaga (liopygia) argyrostoma (Robineau-Desvoidy, 1830). Sci. Rep. 10, 17337. 10.1038/s41598-020-74475-1 33060748 PMC7562915

[B34] Kendrick E. (1963). A mass scale based on CH _2_ = 14.0000 for high resolution Mass spectrometry of organic compounds. Anal. Chem. 35, 2146–2154. 10.1021/ac60206a048

[B35] Khalil S. M. Römpp A. Pretzel J. Becker K. Spengler B. (2015). Phospholipid topography of whole-body sections of the *Anopheles stephensi* mosquito, characterized by high-resolution atmospheric-pressure scanning microprobe matrix-assisted laser desorption/ionization mass spectrometry imaging. Anal. Chem. 87, 11309–11316. 10.1021/acs.analchem.5b02781 26491885

[B36] Khalil S. M. Pretzel J. Becker K. Spengler B. (2017). High-resolution AP-SMALDI mass spectrometry imaging of *Drosophila melanogaster* . Int. J. Mass Spectrom. 416, 1–19. 10.1016/j.ijms.2017.04.001

[B37] Klupczynska A. Pawlak M. Kokot Z. J. Matysiak J. (2018). Application of metabolomic tools for studying low molecular-weight fraction of animal venoms and poisons. Toxins (Basel) 10, 306. 10.3390/toxins10080306 30042318 PMC6116190

[B38] Krijnen K. Blenkinsopp P. Heeren R. M. A. Anthony I. G. M. (2024). Processing next-generation mass spectrometry imaging data: principal component analysis at scale. J. Am. Soc. Mass Spectrom. 35, 3063–3069. 10.1021/jasms.4c00314 39467687 PMC11622226

[B39] Kune C. McCann A. Raphaël L. R. Arias A. A. Tiquet M. Van Kruining D. (2019). Rapid visualization of chemically related compounds using kendrick mass defect as a filter in mass spectrometry imaging. Anal. Chem. 91, 13112–13118. 10.1021/acs.analchem.9b03333 31509388

[B40] La Rocca R. Kune C. Tiquet M. Stuart L. Eppe G. Alexandrov T. (2021). Adaptive pixel mass recalibration for mass spectrometry imaging based on locally endogenous biological signals. Anal. Chem. 93, 4066–4074. 10.1021/acs.analchem.0c05071 33583182

[B41] Laino A. Cunningham M. Costa F. G. Garcia C. F. (2013). Energy sources from the eggs of the wolf spider Schizocosa malitiosa: isolation and characterization of lipovitellins. Comp. Biochem. Physiology - B Biochem. Mol. Biol. 165, 172–180. 10.1016/j.cbpb.2013.04.004 23618789

[B42] Langenegger N. Nentwig W. Kuhn-Nentwig L. (2019). Spider venom: components, modes of action, and novel strategies in transcriptomic and proteomic analyses. Toxins (Basel) 11, 611. 10.3390/toxins11100611 31652611 PMC6832493

[B43] Lim H. Y. Wang W. Wessells R. J. Ocorr K. Bodmer R. (2011). Phospholipid homeostasis regulates lipid metabolism and cardiac function through SREBP signaling in Drosophila. Genes Dev. 25, 189–200. 10.1101/gad.1992411 21245170 PMC3022264

[B44] Lu W. Shi R. Li X. Ma S. Yang D. Shang D. (2024). A review on complete silk gene sequencing and *de novo* assembly of artificial silk. Int. J. Biol. Macromol. 264, 130444. 10.1016/j.ijbiomac.2024.130444 38417762

[B45] Ma L. Xie Q. Du M. Huang Y. Chen Y. Chen D. (2022). Sample preparation optimization of insects and zebrafish for whole-body mass spectrometry imaging. Anal. Bioanal. Chem. 414, 4777–4790. 10.1007/s00216-022-04102-7 35508646

[B46] Ma Q. Wang Z. Xu H. Wei Y. Liang M. (2024). Effects of dietary cholesterol on ovary development and reproductive capacity in Pacific white shrimp broodstock, litopenaeus vannamei. Aquac. Rep. 38, 102346. 10.1016/j.aqrep.2024.102346

[B47] Marc P. Canard A. Ysnel F. (1999). Spiders (araneae) useful for Pest limitation and bioindication. Agric. Ecosyst. Environ. 74, 229–273. 10.1016/S0167-8809(99)00038-9

[B48] Martínez-Gardeazabal J. González de San Román E. Moreno-Rodríguez M. Llorente-Ovejero A. Manuel I. Rodríguez-Puertas R. (2017). Lipid mapping of the rat brain for models of disease. Biochim. Biophys. Acta Biomembr. 1859, 1548–1557. 10.1016/j.bbamem.2017.02.011 28235468

[B49] McCann A. Rappe S. La Rocca R. Tiquet M. Quinton L. Eppe G. (2021). Mass shift in mass spectrometry imaging: comprehensive analysis and practical corrective workflow. Anal. Bioanal. Chem. 413, 2831–2844. 10.1007/s00216-021-03174-1 33517478

[B50] Meyer H. Bossen J. Janz M. Müller X. Künzel S. Roeder T. (2024). Combined transcriptome and proteome profiling reveal cell-type-specific functions of drosophila Garland and pericardial nephrocytes. Commun. Biol. 7, 1424. 10.1038/s42003-024-07062-z 39487357 PMC11530456

[B51] Palmer A. Phapale P. Chernyavsky I. Lavigne R. Fay D. Tarasov A. (2017). FDR-Controlled metabolite annotation for high-resolution imaging mass spectrometry. Nat. Methods 14, 57–60. 10.1038/nmeth.4072 27842059

[B52] Polilov A. A. Makarova A. A. Pang S. Shan Xu C. Hess H. (2021). Protocol for preparation of heterogeneous biological samples for 3D electron microscopy: a case study for insects. Sci. Rep. 11, 4717. 10.1038/s41598-021-83936-0 33633143 PMC7907262

[B53] Propistsova E. A. Makarova A. A. Eskov K. Y. Polilov A. A. (2023). Miniaturization does not change conserved spider anatomy, a case study on spider rayforstia (araneae: anapidae). Sci. Rep. 13, 17219. 10.1038/s41598-023-44230-3 37821480 PMC10567922

[B54] Raghu P. Coessens E. Manifava M. Georgiev P. Pettitt T. Wood E. (2009). Rhabdomere biogenesis in drosophila photoreceptors is acutely sensitive to phosphatidic acid levels. J. Cell Biol. 185, 129–145. 10.1083/jcb.200807027 19349583 PMC2700502

[B55] Rayner S. Vitkauskaite A. Healy K. Lyons K. McSharry L. Leonard D. (2022). Worldwide web: high venom potency and ability to optimize venom usage make the globally invasive noble false widow spider Steatoda nobilis (thorell, 1875) (theridiidae) highly competitive against native European spiders sharing the same habitats. Toxins (Basel) 14, 587. 10.3390/toxins14090587 36136525 PMC9500793

[B56] Römer L. Scheibel T. (2008). The elaborate structure of spider silk: structure and function of a natural high performance fiber. Prion 2, 154–161. 10.4161/pri.2.4.7490 19221522 PMC2658765

[B57] Ryan Wilson (Vector), B. Kimmel (Numbered version), and CC BY 3.0 (2010). Spider figure adapted from the spider book (1912, 1920) by john henry comstock and from biology of spiders (1996) by rainer F. Foelix. Available online at: https://upload.wikimedia.org/wikipedia/commons/3/39/Spider_internal_anatomy-numb.svg.

[B58] Sánchez-Juanes F. Calvo Sánchez N. Belhassen García M. Vieira Lista C. Román R. M. Álamo Sanz R. (2022). Applications of MALDI-TOF mass spectrometry to the identification of parasites and arthropod vectors of human diseases. Microorganisms 10, 2300. 10.3390/microorganisms10112300 36422371 PMC9695109

[B59] Schramm T. Hester A. Klinkert I. Both J. P. Heeren R. M. A. Brunelle A. (2012). ImzML - a common data format for the flexible exchange and processing of mass spectrometry imaging data. J. Proteomics 75, 5106–5110. 10.1016/j.jprot.2012.07.026 22842151

[B60] Shevchenko A. Simons K. (2010). Lipidomics: coming to grips with lipid diversity. Nat. Rev. Mol. Cell Biol. 11, 593–598. 10.1038/nrm2934 20606693

[B61] Smith D. S. Järlfors U. Russell F. E. (1969). The fine structure of muscle attachments in a spider (Latrodectus mactans, fabr.). Tissue Cell 1, 673–687. 10.1016/S0040-8166(69)80040-6 18631493

[B62] Stark W. S. Lin T. Brackhahn D. Christianson J. S. Sun G. Y. (1993). Phospholipids in *drosophila* heads: effects of visual mutants and phototransduction manipulations. Lipids 28, 23–28. 10.1007/BF02536355 8446007

[B63] Steinhauer J. Gijó M. A. Riekhof W. R. Voelker D. R. Murphy R. C. Treisman J. E. (2009). Drosophila lysophospholipid acyltransferases are specifically required for germ cell development. Mol. Biol. Cell 20, 5224–5235. 10.1091/mbc.e09-05-0382 19864461 PMC2793297

[B64] Stoeckli M. Staab D. Schweitzer A. Gardiner J. Seebach D. (2007). Imaging of a beta-peptide distribution in whole-body mice sections by MALDI mass spectrometry. J.Am.Soc.Mass Spectrom. 18, 1921–1924. 10.1016/j.jasms.2007.08.005 17827032

[B65] Stork N. E. (2017). EN63CH03-Stork ARI how many species of insects and other terrestrial arthropods are there on Earth? EN63CH03-Stork ARI. 10.1146/annurev-ento-020117 28938083

[B66] Stutts W. L. Knuth M. M. Ekelöf M. Mahapatra D. Kullman S. W. Muddiman D. C. (2020). Methods for cryosectioning and mass spectrometry imaging of whole-body zebrafish. J. Am. Soc. Mass Spectrom. 31, 768–772. 10.1021/jasms.9b00097 32129621 PMC9375048

[B67] Tiquet M. La Rocca R. Kirnbauer S. Zoratto S. Van Kruining D. Quinton L. (2022). FT-ICR mass spectrometry imaging at extreme mass resolving power using a dynamically harmonized ICR cell with 1ω or 2ω detection. Anal. Chem. 94, 9316–9326. 10.1021/acs.analchem.2c00754 35604839 PMC9260710

[B68] Torres G. Melzer R. R. Spitzner F. Šargač Z. Harzsch S. Gimenez L. (2021). Methods to study organogenesis in decapod crustacean larvae. I. Larval rearing, preparation, and fixation. Helgol Mar. Res. 75, 3. 10.1186/s10152-021-00548-x

[B69] Trabalon M. Garcia C. F. (2021). Transport pathways of hydrocarbon and free fatty acids to the cuticle in arthropods and hypothetical models in spiders. Comp. Biochem. Physiol. B Biochem. Mol. Biol. 252, 110541. 10.1016/j.cbpb.2020.110541 33285310

[B70] Triebl A. Trötzmüller M. Hartler J. Stojakovic T. Köfeler H. C. (2017). Lipidomics by ultrahigh performance liquid chromatography-high resolution mass spectrometry and its application to complex biological samples. J. Chromatogr. B Anal. Technol. Biomed. Life Sci. 1053, 72–80. 10.1016/j.jchromb.2017.03.027 28415015 PMC6013032

[B71] Virmani M. A. Cirulli M. (2022). The role of l-Carnitine in mitochondria, prevention of metabolic inflexibility and disease initiation. Int. J. Mol. Sci. 23, 2717. 10.3390/ijms23052717 35269860 PMC8910660

[B72] Wang Y. Yu Z. Zhang J. Moussian B. (2016). Regionalization of surface lipids in insects. Proc. R. Soc. B Biol. Sci. 283, 20152994. 10.1098/rspb.2015.2994 27170708 PMC4874700

[B73] Wenk M. R. (2005). The emerging field of lipidomics. Nat. Rev. Drug Discov. 4, 594–610. 10.1038/nrd1776 16052242

[B74] Whitehead W. F. Rempel J. G. (1959). A study of the musculature of the black widow spider, Latrodectus mactans (fabr.). Can. J. Zool. 37, 831–870. 10.1139/z59-084

[B75] Wishart D. S. (2019). Metabolomics for investigating physiological and pathophysiological processes. Physiol. Rev. 99, 1819–1875. 10.1152/physrev.00035.2018 31434538

[B76] Wishart D. S. Guo A. C. Oler E. Wang F. Anjum A. Peters H. (2022). HMDB 5.0: the human metabolome Database for 2022. Nucleic Acids Res. 50, D622–D631. 10.1093/nar/gkab1062 34986597 PMC8728138

[B77] Wrońska A. K. Kaczmarek A. Boguś M. I. Kuna A. (2023). Lipids as a key element of insect defense systems. Front. Genet. 14, 1183659. 10.3389/fgene.2023.1183659 37359377 PMC10289264

[B78] Xu H. Chen L. Tong X. L. Hu H. Liu L. Y. Liu G. C. (2022). Comprehensive silk gland multi-omics comparison illuminates two alternative mechanisms in silkworm heterosis. Zool. Res. 43, 585–596. 10.24272/j.issn.2095-8137.2022.065 35726584 PMC9336454

[B79] Xu T. Molday L. L. Molday R. S. (2023). Retinal-phospholipid schiff-base conjugates and their interaction with ABCA4, the ABC transporter associated with Stargardt disease. J. Biol. Chem. 299, 104614. 10.1016/j.jbc.2023.104614 36931393 PMC10127136

[B80] Yang K. Han X. (2016). Lipidomics: techniques, applications, and outcomes related to biomedical sciences. Trends Biochem. Sci. 41, 954–969. 10.1016/j.tibs.2016.08.010 27663237 PMC5085849

[B81] Yang F. Y. Chen J. H. Ruan Q. Q. Saqib H. S. A. He W. Y. You M. S. (2020). Mass spectrometry imaging: an emerging technology for the analysis of metabolites in insects. Arch. Insect Biochem. Physiol. 103, e21643. 10.1002/arch.21643 31667894

[B82] Zhao H. Wang T. (2020). PE homeostasis rebalanced through mitochondria-ER lipid exchange prevents retinal degeneration in Drosophila. PLoS Genet. 16, e1009070. 10.1371/journal.pgen.1009070 33064773 PMC7592913

[B83] Zheng Y. Li Y. Rimm E. B. Hu F. B. Albert C. M. Rexrode K. M. (2016). Dietary phosphatidylcholine and risk of all-cause and cardiovascular-specific mortality among US women and men. Am. J. Clin. Nutr. 104, 173–180. 10.3945/ajcn.116.131771 27281307 PMC4919531

