# Supplementary figures and images for: Organ-resolved lipid mapping in Steatoda nobilis spider model using high-resolution mass spectrometry imaging and Kendrick mass defect analysis
Source: Front Chem. 2025 Sep 3;13:1658546. doi: 10.3389/fchem.2025.1658546 (PMC12442040; doi:10.3389/fchem.2025.1658546)

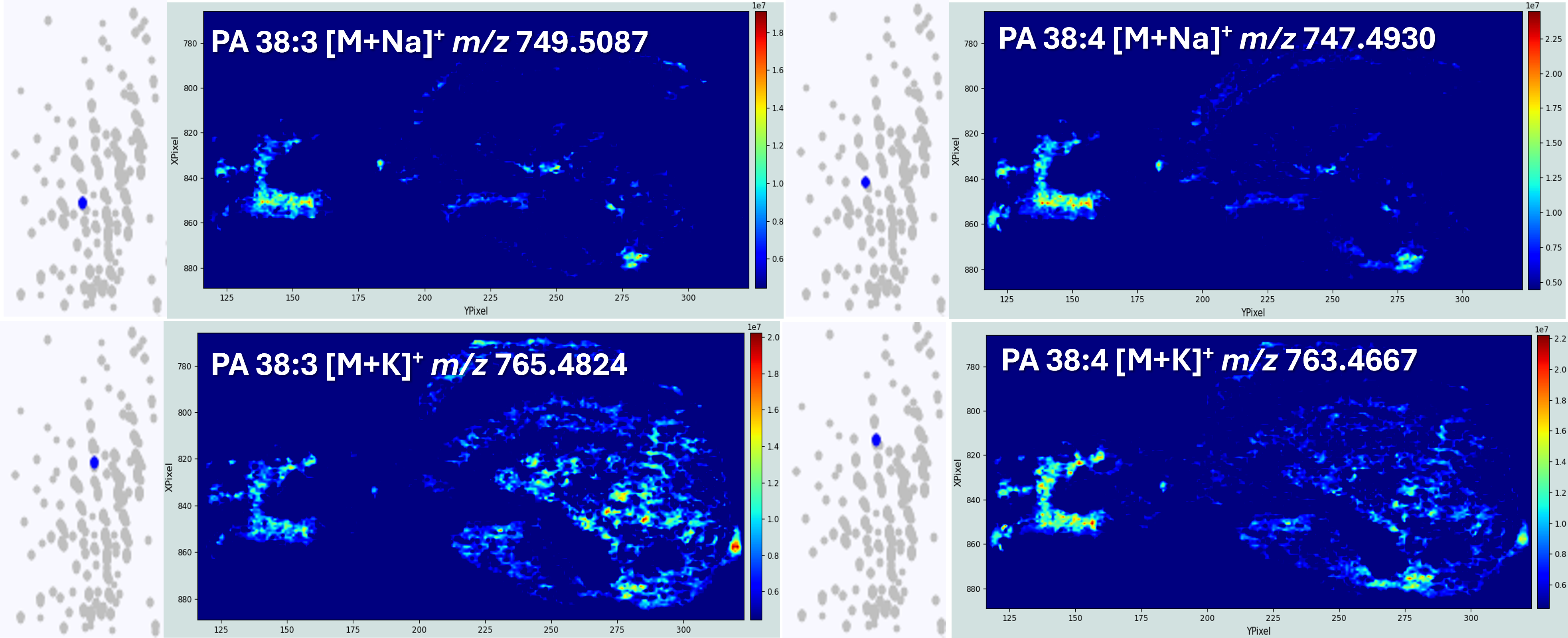

Supplement: Supplementary file 1 [file Image6.tif]

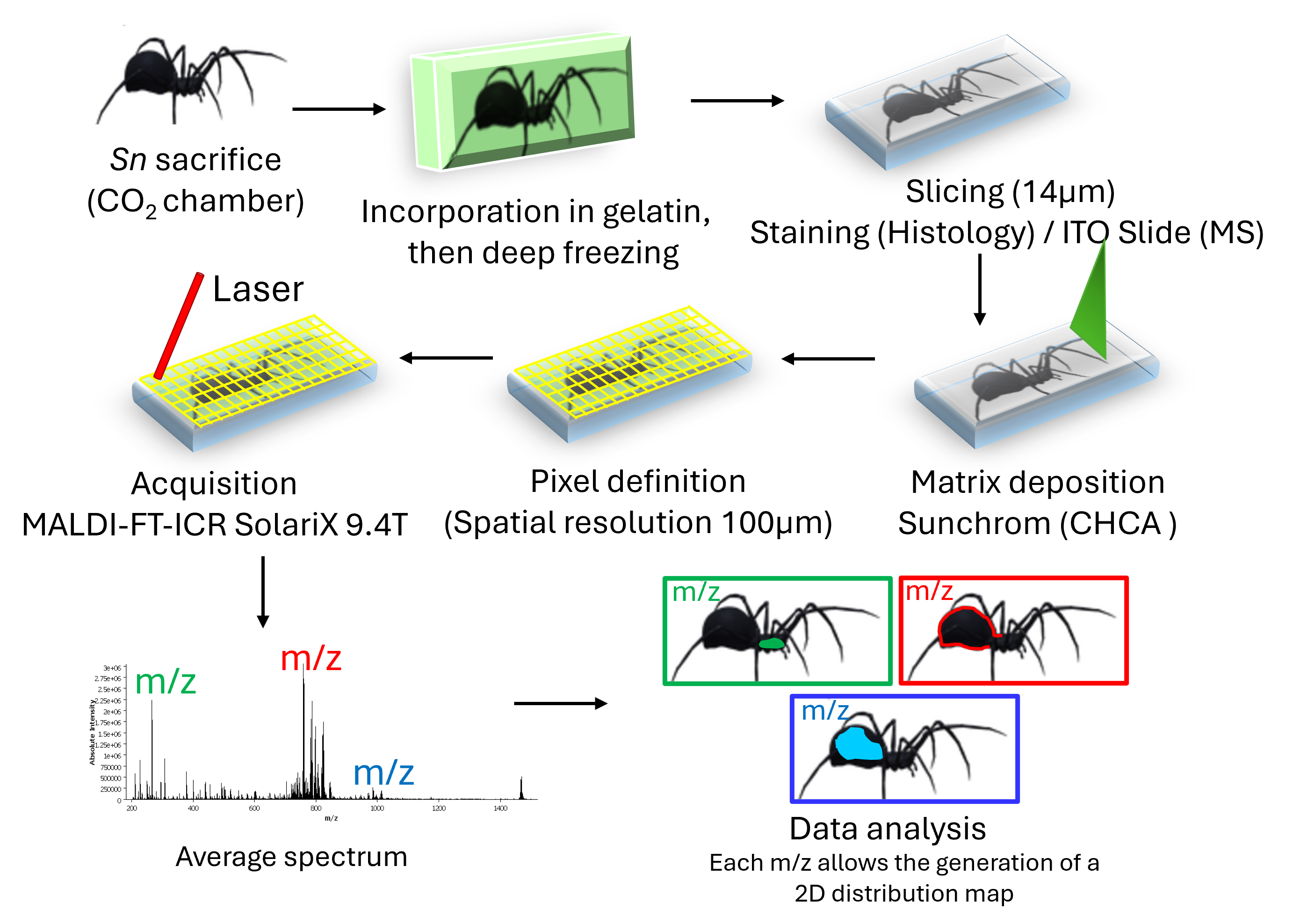

Supplement: Supplementary file 2 [file Image3.tif]

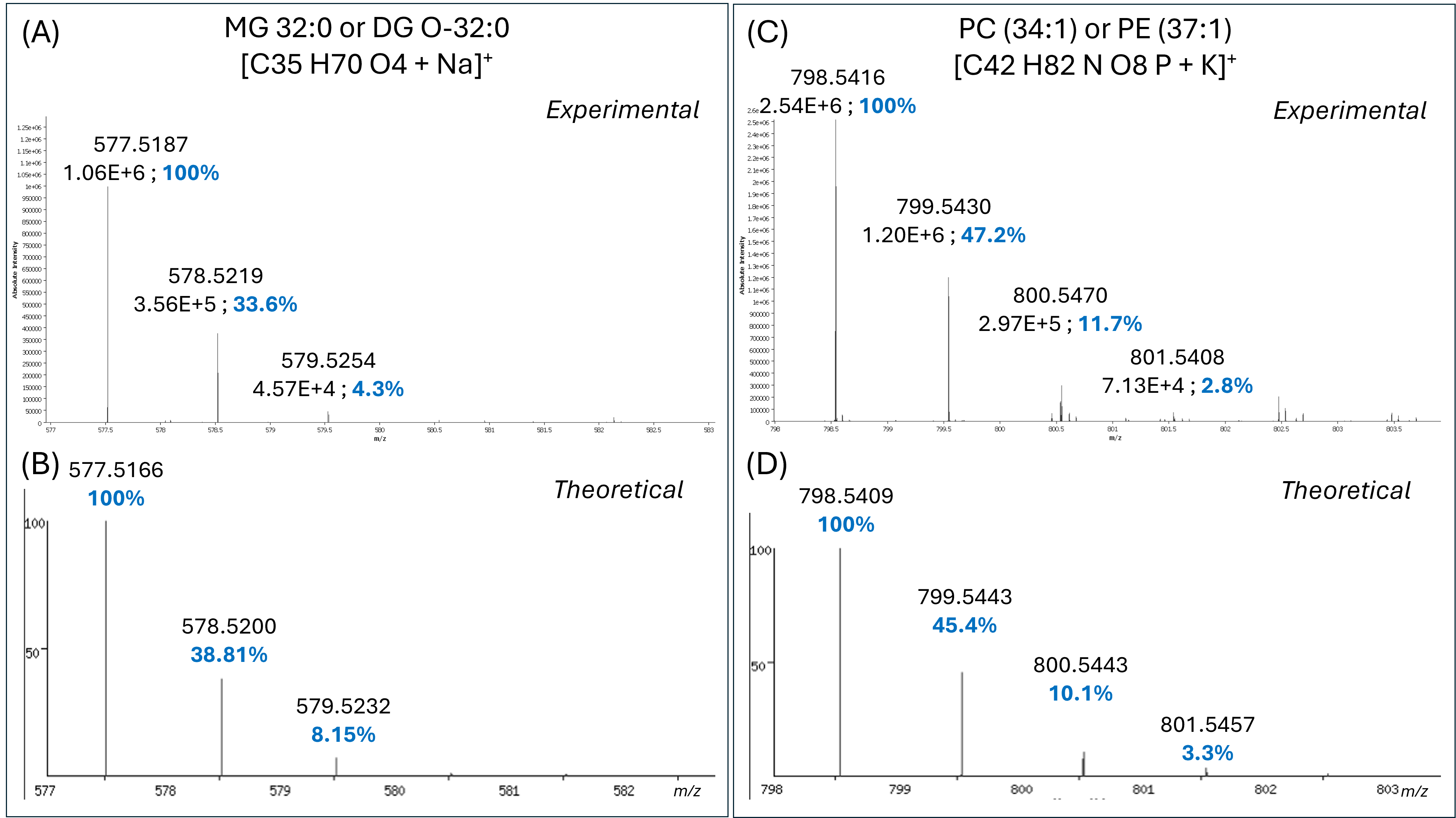

Supplement: Supplementary file 3 [file Image4.tif]

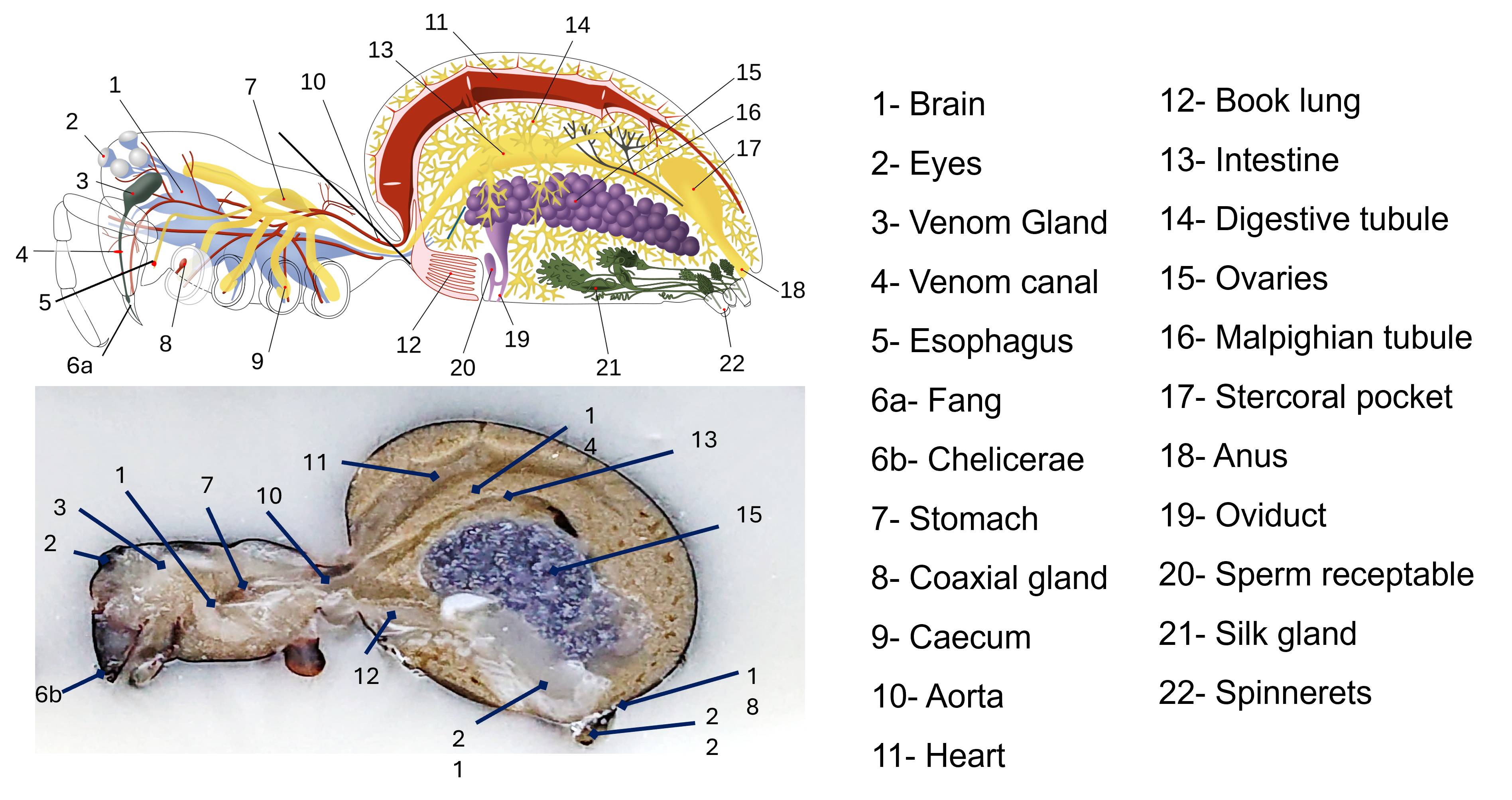

Supplement: Supplementary file 4 [file Image2.tif]

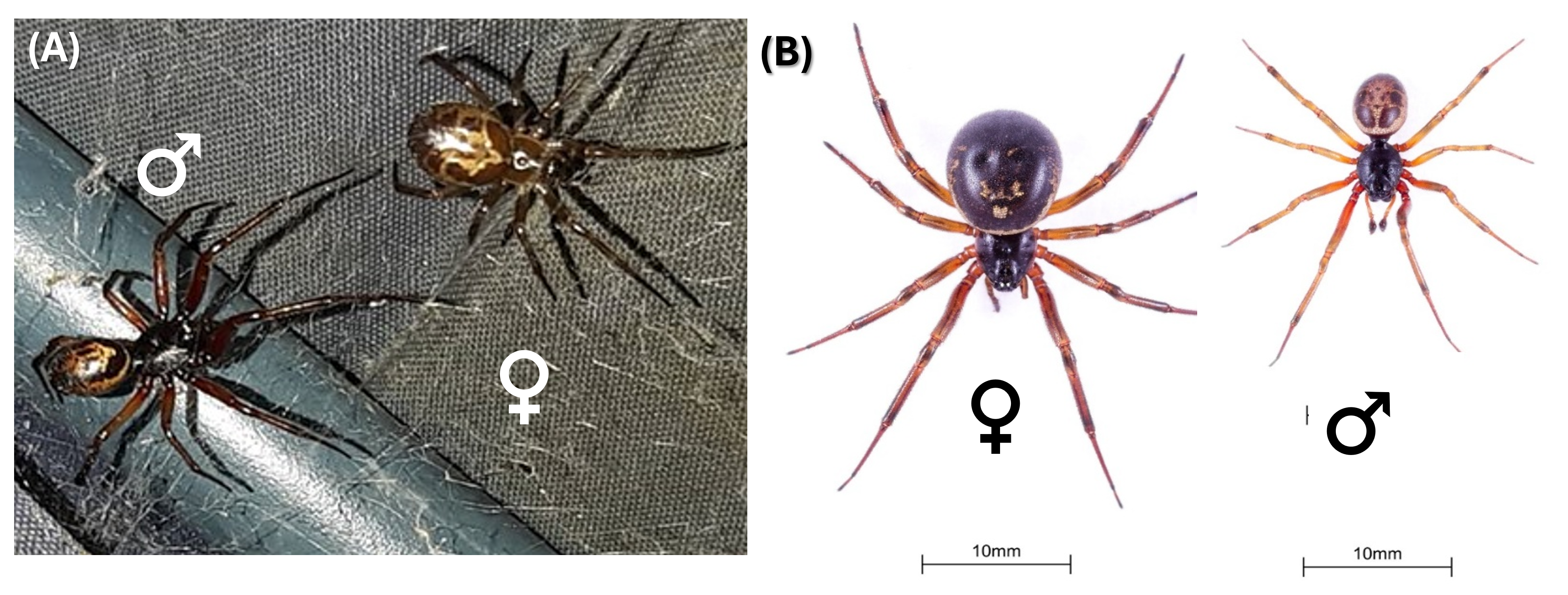

Supplement: Supplementary file 5 [file Image1.tif]

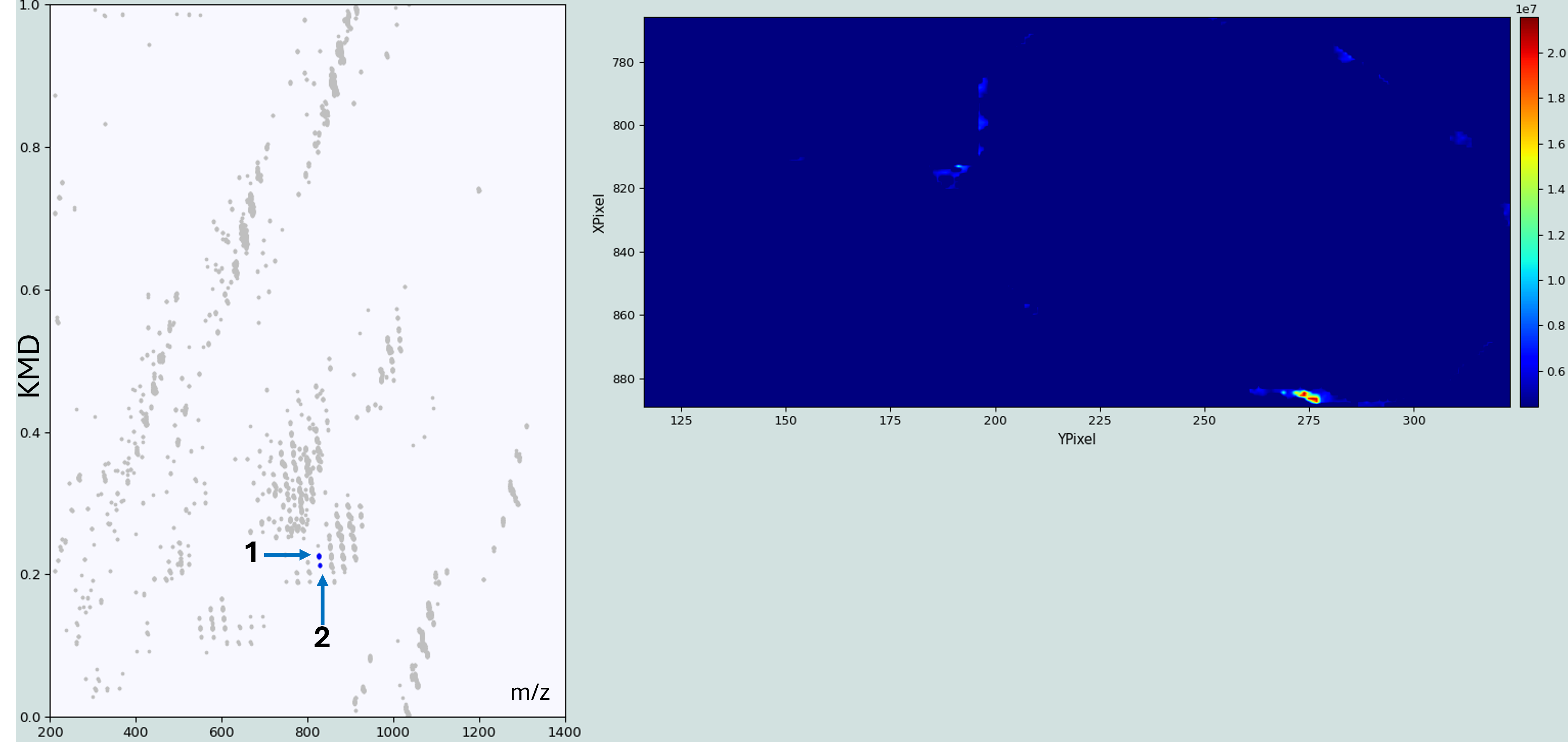

Supplement: Supplementary file 6 [file Image5.tif]
